# AKAP1 Protects Retinal Ganglion Cells and Preserves Vision by Improving Mitochondrial and Synaptic Health in Glaucomatous Neurodegeneration

**DOI:** 10.1002/advs.78349

**Published:** 2026-10-18

**Authors:** Tonking Bastola, Keun‐Young Kim, Ziyao Shen, Guy A. Perkins, Muna Poudel, Yoonjin Lim, Hyejeong Choi, Soo‐Ho Choi, Mark H. Ellisman, Robert N. Weinreb, Won‐Kyu Ju

**Affiliations:** ^1^ Viterbi Family Department of Ophthalmology Viterbi Family Vision Research Center Shiley Eye Institute University of California San Diego La Jolla California USA; ^2^ National Center for Microscopy and Imaging Research Department of Neurosciences University of California San Diego La Jolla California USA; ^3^ Department of Medicine University of California San Diego La Jolla California USA; ^4^ Institute of Engineering in Medicine Shu Chien – Gene Lay Department of Bioengineering University of California San Diego La Jolla California USA

**Keywords:** biology, cell biology, mitochondrial biogenesis, mitochondrion, neurodegeneration, neuroprotection, neuroscience, retinal ganglion, synaptophysin

## Abstract

Glaucoma, a leading cause of irreversible blindness, is characterized by progressive loss of retinal ganglion cells (RGCs) and lacks effective neuroprotective therapies. Despite evidence implicating mitochondrial dysfunction in glaucoma, the mechanisms linking elevated intraocular pressure, synaptic dysfunction, mitochondrial failure, and RGC death remain incompletely understood. Here we identify the mitochondrial scaffold protein A‐kinase anchoring protein 1 (AKAP1) as a critical regulator of RGC resilience in glaucoma. AKAP1 expression is reduced in glaucomatous retinas and correlates with disrupted mitochondrial dynamics, oxidative stress, synaptic impairment, and visual decline. AAV2‐mediated restoration of AKAP1 preserves RGC survival and visual function in two independent experimental glaucoma models. These data suggest that retinal AKAP1 promotes mitochondrial fusion, cristae integrity, and bioenergetic capacity in the inner retina and in stressed cultured RGCs, and is accompanied by increased mitochondrial content in presynaptic terminals of the inner plexiform layer. Retinal AKAP1 is further associated with higher PKA abundance, normalized CaMKII and synapsin phosphorylation, preserved synaptophysin, and lower complement C1q and glial activation markers; these associations do not, by themselves, establish causality. These findings identify AKAP1 as a candidate regulator of mitochondrial and synaptic homeostasis and highlight AAV2‐AKAP1 gene therapy as a promising neuroprotective strategy for vision preservation in glaucoma.

## Graphical abstract

Intravitreal AAV2‐mediated AKAP1 restoration enhances mitochondrial fusion and biogenesis, improves bioenergetic capacity, and preserves mitochondrial integrity in glaucomatous RGCs. AKAP1 stabilizes presynaptic structure, maintaining synaptic protein expression, and limiting complement‐associated neuroinflammation. These coordinated effects protect RGCs, preserve the central visual pathway, and restore visual function. Together, AKAP1 emerges as a multifaceted regulator of mitochondrial and synaptic homeostasis and as a promising therapeutic strategy for preserving vision in glaucoma. The graphic abstract includes graphical elements created by BioRender (www.biorender.com).

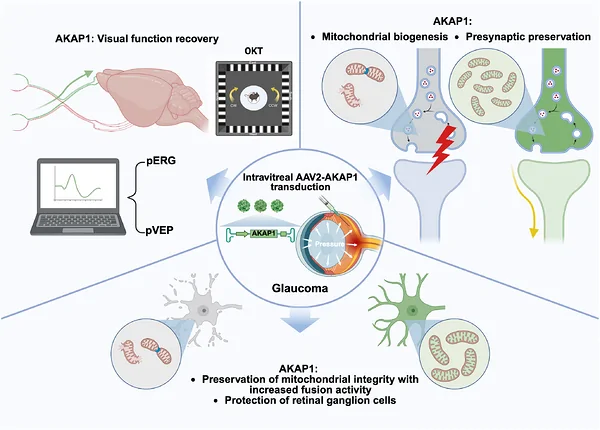

## 1 Introduction

Glaucoma, a multifactorial neurodegenerative disease and a leading cause of irreversible blindness worldwide, is characterized by progressive optic nerve (ON) degeneration and retinal ganglion cell (RGC) loss. Although elevated intraocular pressure (IOP) is a major risk factor, lowering IOP slows but does not halt disease progression, underscoring the need for IOP‐independent neuroprotective strategies. Increasing evidence indicates that mitochondrial dysfunction is a central driver of glaucomatous neurodegeneration, particularly through impaired oxidative phosphorylation (OXPHOS), which is associated with cytochrome c oxidase subunit I polymorphisms [1, 2, 3, 4, 5]. Despite this, the molecular mechanisms linking elevated IOP, synaptic dysfunction, mitochondrial failure, and RGC degeneration remain incompletely understood [2, 6, 7].

A‐kinase anchoring protein 1 (AKAP1) is a mitochondrial scaffold protein localized to the outer mitochondrial membrane (OMM), where it coordinates signaling pathways that regulate mitochondrial dynamics, bioenergetics, and cell survival [2, 8, 9]. AKAP1 anchors protein kinase A (PKA) and modulates key processes, including mitochondrial fission–fusion balance, calcium signaling, and reactive oxygen species production. AKAP1/PKA signaling components are broadly expressed across multiple retinal layers, including RGCs, bipolar cells, and amacrine cells, suggesting a conserved role for this pathway in mitochondrial regulation across diverse retinal neuron types [8]. Notably, AKAP1 has been shown to confer neuroprotection in models of cerebral ischemia by maintaining respiratory chain activity, inhibiting dynamin‐related protein 1 (DRP1)‐mediated mitochondrial fission, and reducing oxidative stress [10]. In the retina, reduced AKAP1 expression has been associated with increased calcineurin (CaN) activity, decreased DRP1 phosphorylation at serine 637, disrupted mitochondrial dynamics, and enhanced RGC vulnerability [8, 11].

Beyond mitochondrial dysfunction, accumulating evidence suggests that early synaptic alterations precede RGC soma loss in glaucoma [12, 13, 14]. Synapses within the inner plexiform layer (IPL), particularly those of bipolar, amacrine, and RGCs, are highly metabolically active and therefore especially susceptible to oxidative stress and mitochondrial impairment [13, 14, 15, 16]. Synaptic pruning and dysfunction further compromise neuronal communication, accelerating neurodegeneration. Given that mitochondrial dynamics are critical for synaptic maintenance and function [17, 18], disruption of AKAP1‐mediated signaling may contribute not only to mitochondrial defects but also to early synaptic failure in glaucoma.

Here, we investigate the role of AKAP1 in regulating mitochondrial integrity, synaptic activity, and RGC survival in experimental models of glaucoma. We demonstrate that AKAP1 expression is reduced in glaucomatous retinas and that its restoration via adeno‐associated virus serotype 2 (AAV2) preserves mitochondrial function, synaptic structure, and visual performance. Mechanistically, AKAP1 enhances mitochondrial fusion, bioenergetic capacity, and resistance to oxidative stress while maintaining synaptic integrity by modulating PKA and CaMKII signaling pathways. These findings identify AKAP1 as a central regulator of mitochondrial and synaptic homeostasis and highlight its potential as a therapeutic target for neuroprotection in glaucomatous neurodegeneration.

## 2 Results

### 2.1 AKAP1 Protects RGCs and Preserves Visual Function in Glaucomatous D2 Mice

To determine whether AKAP1 restoration confers neuroprotection in glaucoma, we intravitreally injected AAV2‐AKAP1 or AAV2‐Null into 5‐month‐old DBA/2J (D2) mice [19, 20], and evaluated outcomes at 10 months of age (Figure 1A). Age‐matched D2‐*Gpnmb^+^* (D2G‐Null) mice were used as non‐glaucomatous controls [21]. As expected, D2 mice exhibited elevated IOP, and both AKAP1 and the RGC marker RNA‐binding protein with multiple splicing (RBPMS) were significantly reduced in glaucomatous retinas (Figure 1B and Figure S1A,B). To ensure experimental consistency, only D2 mice with IOP values between 20 and 35 mmHg were included in subsequent analyses (Figure 1B and Figure S1A,B). AAV2‐mediated AKAP1 delivery effectively produced robust retinal AKAP1 expression, as confirmed by His‐tag detection and Western blot analysis, and also increased PKA levels in the retina (Figure 1C–I and Figure S1C–E). Immunohistochemical analyses further demonstrated recovery of AKAP1 and PKA expression within the inner retina, including the ganglion cell layer (GCL) (Figure 1F–I). Furthermore, PKA expression in AKAP1*^−/−^* retina showed a decreasing pattern by Western blot analysis, although the difference did not reach statistical significance, whereas immunohistochemical analysis revealed a significant reduction in the GCL compared with wild‐type (WT) retina (Figure S2A–C), suggesting that AKAP1 is associated with regulation of PKA signaling components in the GCL. Importantly, AKAP1 restoration significantly preserved RGC survival in both middle and peripheral retina compared with D2‐Null mice (Figure 1J,K; see also Table S1). Consistent with structural preservation, AKAP1 restoration improved axonal transport and retinal connectivity to the brain. Cholera toxin subunit B (CTB) labeling revealed enhanced anterograde transport to the superior colliculus (SC), indicating preserved retina‐to‐brain connectivity (Figure 1L,M). Functional analyses further demonstrated that AKAP1 restoration significantly improved pattern electroretinogram (pERG) and pattern visual evoked potential (pVEP) amplitudes, whereas pVEP latency remained unchanged (Figure 1N,O). Together, these findings demonstrate that AAV2‐mediated AKAP1 expression in the inner retina preserves RGC survival, structural connectivity, and visual function in glaucomatous degeneration (Figure 1P).

**FIGURE 1:**
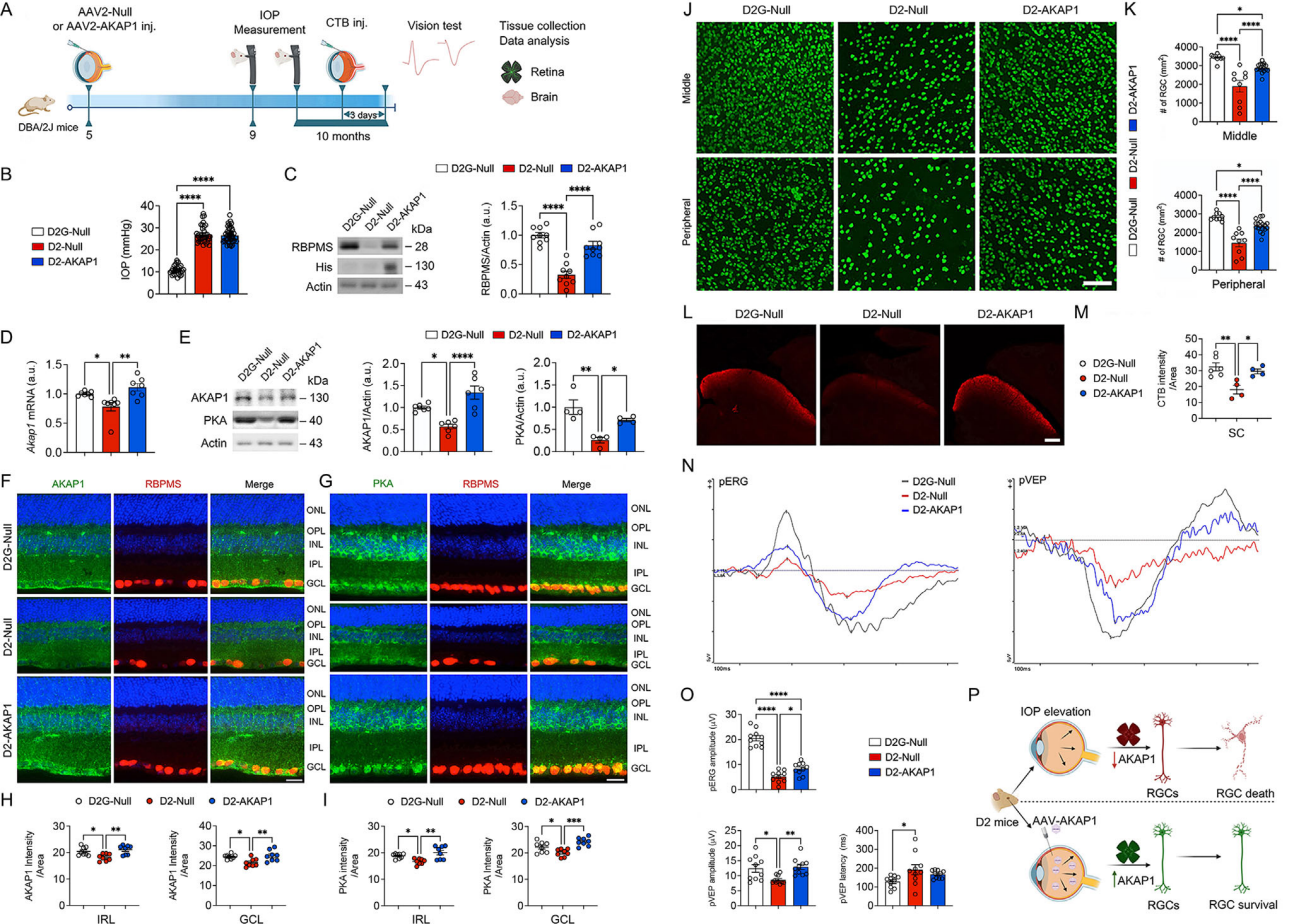
AKAP1 protects RGCs and preserves visual function in glaucomatous D2 retinas. (A) Experimental schematic and timeline of AAV injection, IOP measurement, tissue collection, and data analysis in D2G and D2 mice (*n* = 20 mice per group). (B) IOP measurement in 10‐month‐old D2G‐Null, D2‐Null, and D2‐AKAP1 mice (*n* = 20 mice per group). (C) Representative image of a Western blot and densitometry graph for RBPMS and His expression in the retinas (*n* = 9 retinas from 9 mice per group). (D) Quantitative PCR for AKAP1 gene expression in the retinas (*n* = 7 retinas from 7 mice per group). (E) Representative image of a Western blot and densitometry graph for AKAP1 and PKA expression in the retinas (*n* = 4 to 6 retinas from 4 to 6 mice per group). (F, G) Representative retinal images for AKAP1 (green), PKA (green), and RBPMS (red) immunoreactivities. (H, I) Quantitative fluorescent intensity of AKAP1 immunoreactivity in the inner retinal layer (IRL: IRL includes INL, IPL, and GCL) and GCL (*n* = 8 retina sections from 3 mice per group). (J) Representative retina whole‐mounted images for RBPMS (green)‐positive RGCs in the middle and peripheral areas of the retina. (K) Quantitative analysis of RGC numbers in the middle and peripheral areas of the retina (*n* = 9 to 18 retina whole mounts from 9 to 18 mice per group). (L) Representative images for CTB labeling in the SC. (M) Quantitative analysis of CTB intensity in the SC (*n* = 4 to 6 SC sections from 3 mice per group). (N) Representative recording graphs for pERG and pVEP measurements. (O) Quantitative analysis of pERG amplitude, pVEP amplitude, and pVEP latency (*n* = 10 mice per group). Note that AKAP1 expression restores visual function in glaucomatous D2 mice. (P) Schematic for the effect of sustained AKAP1 expression on glaucomatous retina. Error bars represent SEM. Statistical analysis was performed using one‐way ANOVA and Tukey's multiple comparisons test. ^*^ *p* < 0.05, ^**^ *p* < 0.01, and ^****^ *p* < 0.0001. Scale bars, 20 µm (F, G), 50 µm (J), 100 µm (L). Panels (A, P) include graphical elements created by BioRender (www.biorender.com).

### 2.2 AKAP1 Protects RGCs and Preserves Visual Function in Microbead‐Induced Glaucoma

To further validate the neuroprotective effects of AKAP1 in an inducible glaucoma model, we employed a microbead (MB)‐induced ocular hypertension (OHT) model [22, 23]. Three‐month‐old C57BL/6J mice received intravitreal injections of AAV2‐AKAP1 or AAV2‐Null 3 weeks prior to MB injection (Figure 2A). Sustained elevation of IOP (20–30 mmHg) was achieved and did not differ significantly between experimental groups (Figure 2B), ensuring that observed effects were independent of pressure variability. MB‐induced OHT resulted in significant RGC loss in Null‐MB mice compared with control mice (Figure 2C,D; see also Table S2). In contrast, AKAP1 restoration markedly preserved RGC numbers despite sustained IOP elevation (Figure 2C,D; see also Table S2). Functional analyses revealed that MB‐induced OHT significantly reduced pERG and pVEP amplitudes and prolonged pVEP latency. Notably, AKAP1 restoration significantly improved pERG and pVEP amplitudes and normalized pVEP latency (Figure 2E,F). These results confirm that AKAP1 exerts robust neuroprotective effects across both spontaneous and inducible glaucoma models.

**FIGURE 2:**
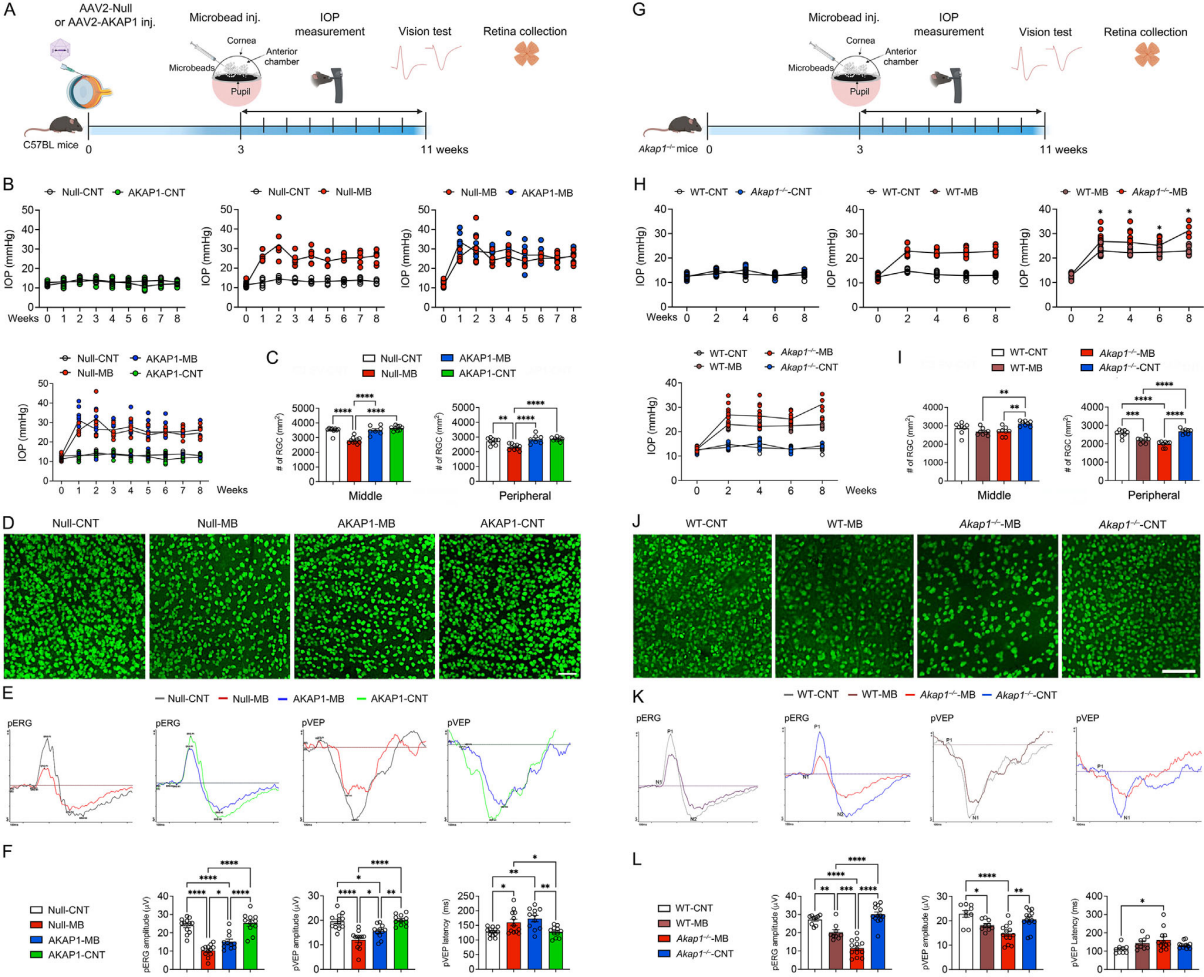
AKAP1 protects RGCs and preserves visual function, but AKAP1 loss worsens RGC function in glaucomatous MB‐OHT retinas. (A) Experimental schematic and timeline of AAV injection, IOP measurement, tissue collection, and data analysis in glaucomatous MB mice (*n* = 12 to14 mice per group). (B) Monitoring of IOP elevation in CNT and glaucomatous MB mice (*n* = 12 to 14 mice per group). (C) Quantitative analysis of RGC number in the middle and peripheral areas of the retina (*n* = 7 to 10 retina whole mounts from 7 to 10 mice per group). (D) Representative whole‐mounted retina images for RBPMS (green) immunoreactivity in the middle area. (E) Representative recording graphs for pERG and pVEP measurements. (F) Quantitative analysis of pERG amplitude, pVEP amplitude, and pVEP latency (*n* = 12 to 14 mice per group). Note that AKAP1 expression restores visual function in glaucomatous MB mice. (G) Experimental schematic and timeline of MB injection, IOP measurement, tissue collection, and data analysis in glaucomatous AKAP1*^−/−^*‐MB mice (*n* = 9 mice per group). (H) Monitoring of IOP elevation in AKAP1*^−/−^*‐MB mice (*n* = 9 to 12 mice per group). (I) Quantitative analysis of RGC number in the middle and peripheral areas of the retina (*n* = 8 retina whole mounts per group). (J) Representative whole‐mounted retina images for RBPMS (green) immunoreactivity in the middle area from AKAP1*^−/−^*‐MB mice. (K) Representative recording graphs for pERG and pVEP measurements. (L) Quantitative analysis of pERG amplitude, pVEP amplitude, and pVEP latency (*n* = 9 to 13 mice per group). Note that AKAP1 deficiency worsens RGC dysfunction in glaucomatous MB mice. Error bars represent SEM. Statistical analysis was performed using one‐way ANOVA and Tukey's multiple comparisons test. ^*^ *p* < 0.05, ^**^ *p* < 0.01, ^***^ *p* < 0.001, and ^****^ *p* < 0.0001. Scale bars: 50 µm (D) and 100 µm (J). Panels (A, G) include graphical elements created by BioRender (www.biorender.com).

### 2.3 AKAP1 Deficiency Exacerbates RGC Dysfunction Under Glaucomatous Stress

To further assess the role of endogenous AKAP1, we examined the effects of AKAP1 deficiency using AKAP1*^−/−^* mice subjected to MB‐induced OHT (Figure 2G). IOP elevation following MB injection was similar between WT‐MB and AKAP1*^−/−^*‐MB mice, with AKAP1*^−/−^*‐MB mice showing a slightly higher mean IOP (Figure 2H). This difference reached statistical significance. Although its magnitude was small, we did not formally test whether it accounts for the functional phenotype, and we therefore cannot exclude that the modestly higher IOP in AKAP1‐deficient mice contributed, at least in part, to the reduced pERG response. While MB injection reduced RGC numbers in WT mice compared with controls, AKAP1 deficiency did not further decrease RGC counts (Figure 2I,J; see also Table S3). However, functional assessment revealed a significant exacerbation of RGC dysfunction in AKAP1*^−/−^*‐MB mice. Specifically, pERG amplitudes were further reduced compared with WT‐MB mice, whereas pVEP parameters remained comparable between groups (Figure 2K,L). These findings suggest that endogenous AKAP1 contributes to maintaining RGC functional integrity under glaucomatous stress, even in the absence of additional cell loss, while acknowledging that a small pressure difference between genotypes may also have contributed.

### 2.4 AKAP1 Promotes Mitochondrial Fusion and Preserves Mitochondrial Dynamics in Glaucomatous D2 Retinas

We next investigated whether AKAP1 restoration modulates mitochondrial dynamics in glaucomatous retinas. CaN, a regulator of DRP1 activation [8, 11], was significantly elevated in D2‐Null retinas but normalized following AKAP1 restoration (Figure 3A). Consistently, *Drp1* mRNA expression was increased in glaucomatous retinas and reduced upon AKAP1 expression (Figure 3B). Western blot analysis revealed decreased total DRP1 protein levels and increased phosphorylation at serine 637 (pDRP1 S637), a modification associated with inhibition of mitochondrial fission, whereas phosphorylation at serine 616 remained unchanged (Figure 3C). Immunohistochemistry confirmed reduced DRP1 expression in the IPL and within RGCs and the GCL following AKAP1 restoration (Figure 3D,E). In parallel, expression of the mitochondrial fusion protein optic atrophy type 1 (OPA1), which was reduced in glaucomatous retinas, was increased following AKAP1 overexpression (Figure 3F,G). Further, we quantified the OPA1‐S/L ratio. D2‐Null significantly reduced the S/L ratio compared with D2G‐Null controls (Figure 3G), indicating that OPA1 processing is altered under these conditions. AKAP1 restoration increased total OPA1 and L‐OPA1 levels without a corresponding change in S‐OPA1, and the S/L ratio did not differ significantly from that in D2‐Null (Figure 3G). These results indicate that AKAP1 primarily preserves OPA1 abundance and the fusion‐competent long form, rather than acting by modulating OPA1 proteolytic processing. However, mitofusin (MFN) 1 and MFN2 protein expression remained comparable across groups (Figure 3H,I). Together, these findings suggest that retinal AKAP1 expression improves mitochondrial homeostasis in the glaucomatous retina by promoting mitochondrial fusion activity through modulation of DRP1 phosphorylation and OPA1 expression, thereby preserving mitochondrial integrity under glaucomatous stress.

**FIGURE 3:**
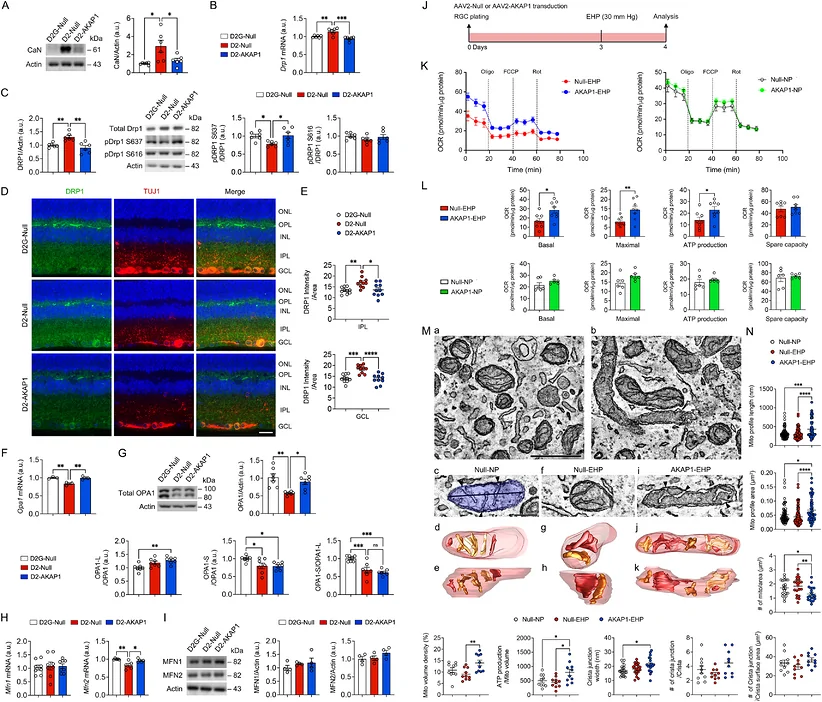
AKAP1 enhances mitochondrial fusion activity and energy production in glaucomatous RGCs. (A) Representative image of a Western blot and densitometry graph for CaN expression in the retinas of D2G‐Null, D2‐Null, and D2‐AKAP1 mice (*n* = 6 retinas from 6 mice per group). (B) Quantitative PCR for *Drp1* gene expression in the retinas. (C) Representative image of a Western blot and densitometry graph for total DRP1, phospho‐DRP1 (S637), and phospho‐DRP1 (S616) in the retinas (*n* = 6 to 8 retinas from 6 to 8 mice per group). (D) Representative retinal images for DRP1 (green) and TUJ1 (red) immunoreactivities. (E) Quantitative fluorescent intensity of DRP1 immunoreactivity in the GCL of the retinas (*n* = 10 retina sections from 3 mice per group). (F) Quantitative PCR for *Opa1* gene expression in the retinas (*n* = 3 retinas from 3 mice per group). (G) Representative image of a Western blot and densitometry graph for total OPA1, OPA1‐S form, OPA1‐L form, and OPA1‐S/L ratio in the retinas (*n* = 7 retinas from 7 mice per group). (H) Quantitative PCR for *Mfn1*/*2* gene expression (*n* = 5 to 8 retinas per group). (I) Representative image of a Western blot and densitometry graph for MFN1 and MFN2 in the retinas of (*n* = 4 retinas from 4 mice per group). (J) Experimental schematic and timeline of AAV2 transduction and data analysis in RGCs under elevated HP conditions in vitro. (K) Representative graphs for OCR changes in RGCs under elevated HP conditions. (L) Quantitative analysis of OCR parameters: basal respiration, maximal respiration, ATP production, and spare capacity in RGCs under elevated HP conditions (*n* = 6 to 8 biological replicates per group). (M) Slice through an EM tomography volume of a null‐EHP RGC showing a large number (11) of small mitochondria (a). Slice through an EM tomography volume of an AKAP1‐EHP RGC, showing, in comparison to panel a, a smaller number (7) of larger mitochondria, also with a higher mitochondrial volume fraction of the cytoplasm (b). Slice through the middle of an EM tomography volume of a Null‐NP mitochondrion (c). Three crista junctions are seen (arrowheads), with 1 showing a larger width. An example of a mitochondrial profile length measurement is shown by the double arrow. An example of a mitochondrial profile area measurement is indicated by the blue shading. Top view of the surface‐rendered mitochondrial volume after membrane segmentation (d). Side view (e). All the cristae have a lamellar shape. Slice through the middle of an EM tomography volume of a Null‐EHP mitochondrion (f). The crista junction (arrowhead) is wider than 2 of those shown in (a). Top view of the surface‐rendered volume (g). The crista at the top shows a twist, further noted in the side view (h). Slice through the middle of an EM tomography volume of an AKAP1‐elevated HP mitochondrion (i). The 3 crista junctions (arrowheads) shown are wider than 2 of those in (a), especially the one at the bottom right. The matrix is denser (more condensed) than in (a) and (d), suggesting increased metabolism, which is also consistent with wider crista junctions. The top view of the surface‐rendered volume shows that the cristae mostly fill the mitochondrial interior, heightening the crista density (j). Side view showing another perspective of the twisting of several cristae (k). (*N*) Measurements for mitochondrial profile length (*n* = 20 mitochondria per population), profile area (*n* = 20 mitochondria per condition), number per unit area (*n* = 10 mitochondria per condition), volume density (*n* = 10 mitochondria per condition), as well as rate of ATP generation per mitochondrial volume (*n* = 10 mitochondria per condition), crista junction width (*n* = 20 mitochondria per condition), number of crista junction per crista (*n* = 10 mitochondria per condition), and number of crista junction per crista surface area (*n* = 10 mitochondria per condition). The number of replicates was 10 independent, sparsely spaced, primary RGCs, 5 each from 2 plates per condition for all measurements. Bars represent SEM. Statistical analysis was performed using unpaired Student's *t*‐test or one‐way ANOVA and Tukey's multiple comparisons test. ^*^ *p* < 0.05, ^**^ *p* < 0.01, ^***^ *p* < 0.00,1 and ^****^ *p* < 0.0001. Scale bars: 20 µm (*D*) and 200 nm (*M*). NP, normal pressure; HP, hydrostatic pressure.

### 2.5 AKAP1 Enhances Mitochondrial Respiration, Fusion Activity, and ATP Production in RGCs Under Elevated Pressure

To further characterize the functional consequences of AKAP1‐mediated mitochondrial regulation, primary RGCs were transduced with AAV2‐AKAP1 and subjected to elevated hydrostatic pressure (HP) (Figure 3J). GFP expression confirmed efficient transduction in TUJ1‐positive RGCs (Figure S3A,B), and increased AKAP1 and RBPMS expression was observed following transduction (Figure S3C). Under HP conditions, AKAP1 significantly preserved cell viability and reduced cytotoxicity, as assessed by the 3‐[4,5‐dimethylthiazol‐2‐yl]‐2,5‐diphenyltetrazolium bromide (MTT) and lactate dehydrogenase (LDH) assays (Figure S3D). Seahorse analysis demonstrated that AKAP1 enhanced mitochondrial respiration, including basal, maximal, and ATP‐linked oxygen consumption rates (OCR), whereas spare respiratory capacity remained unchanged (Figure 3K,L). Ultrastructural analysis using EM tomography revealed that AKAP1‐expressing RGCs exhibited elongated mitochondria with increased mitochondrial profile length, area, and volume density, and increased crista density and crista junction width. Also observed was reduced mitochondrial number, but there was no difference in the number of crista junctions per crista or crista surface area (Figure 3M,N; see also Movies S1–S3). These structural features were associated with increased modeled ATP production per mitochondrial volume, consistent with enhanced mitochondrial fusion activity and bioenergetic capacity.

### 2.6 AKAP1 Attenuates AMPK and p38 Activation, Oxidative Stress, and Apoptosis in Glaucomatous D2 Retinas

We next examined whether AKAP1 modulates stress signaling pathways associated with glaucomatous degeneration. Phosphorylated 5' AMP‐activated protein kinase (pAMPK) levels were significantly elevated in D2‐Null retinas but markedly reduced following AKAP1 restoration (Figure 4A–C). Similarly, phosphorylated p38 mitogen‐activated protein kinase (p38‐MAPK; pp38), a key mediator of oxidative stress signaling, was increased in glaucomatous retinas and attenuated by AKAP1 expression (Figure 4D–F). Markers of oxidative stress and apoptosis, including SOD2, BAX, and BIM, were upregulated in D2‐Null retinas, whereas the anti‐apoptotic protein Bcl‐xL was reduced (Figure 4G,H). AKAP1 restoration reversed these changes, indicating reduced oxidative stress and apoptotic signaling. These findings demonstrate that AKAP1 suppresses stress‐activated pathways and is associated with enhanced RGC survival.

**FIGURE 4:**
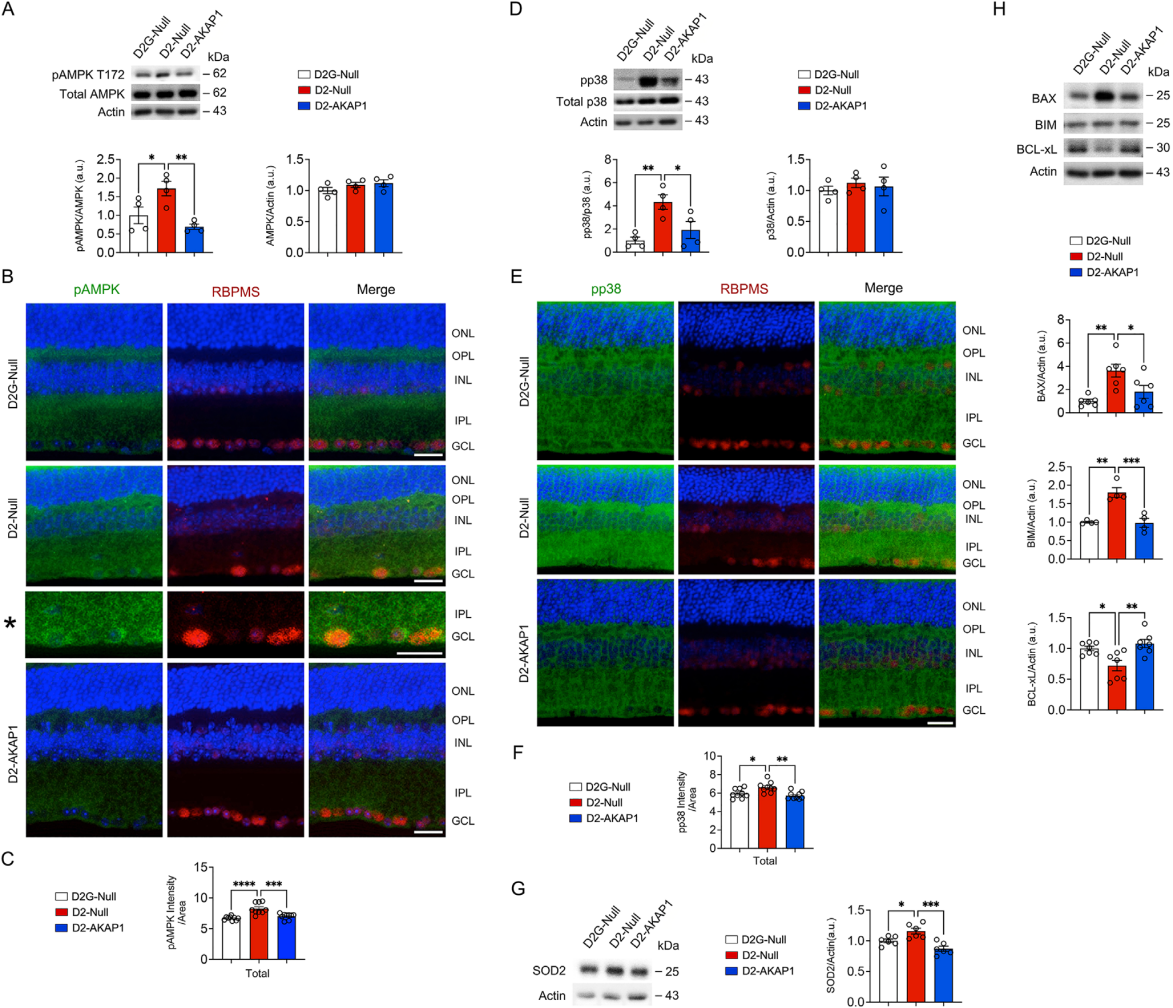
AKAP1 inhibits AMPK and p38 activation, oxidative stress, and apoptosis in glaucomatous D2 retinas. (A) Western blot analysis for phospho‐AMPK (pAMPK) and total AMPK expression in the retinas (*n* = 4 retinas from 4 mice per group). (B) Representative retinal images for pAMPK (green) and RBPMS (red) immunoreactivities. Asterisk (*) indicates high magnification for the representative image (pAMPK and RBPMS) for the D2‐Null retina. (C) Quantitative fluorescent intensity of pAMPK immunoreactivity in the total retinal layer that includes OPL, ONL, INL, IPL, and GCL (*n* = 9 retinal sections from 3 mice per group). (D) Western blot analysis for phospho‐p38 (pp38) and total p38 expression in the retinas (*n* = 4 retinas from 4 mice per group). (E) Representative retinal images for pp38 (green) and RBPMS (red) immunoreactivities. (F) Quantitative fluorescent intensity of pp38 immunoreactivity in the total retinal layer that includes OPL, ONL, INL, IPL, and GCL (*n* = 9 retinal sections from 3 mice per group). (G) Western blot analysis for SOD2 expression in the retinas (*n* = 6 retinas from 6 mice per group). (H) Western blot analysis for BAX, BIM, and BcL‐xL expression in the retinas (*n* = 4 to 7 retinas from 4 to 7 mice per group). Error bars represent SEM. Statistical analysis was performed using one‐way ANOVA and Tukey's multiple comparisons test. ^*^ *p* < 0.05, ^**^ *p* < 0.01, ^***^ *p* < 0.001 and ^****^ *p* < 0.0001. Scale bars, 20 µm.

### 2.7 AKAP1 Protects RGCs From Oxidative Stress‐Induced Mitochondrial Dysfunction

To directly assess the protective effects of AKAP1 under oxidative stress, primary RGCs were treated with paraquat (PQ) (Figure S4A). AKAP1 overexpression significantly increased *Akap1* mRNA levels (Figure S4B) and preserved AKAP1 and RBPMS protein expression under PQ treatment (Figure S4C). PQ exposure reduced cell viability and increased cytotoxicity in control RGCs, whereas AKAP1 overexpression significantly preserved viability and reduced cell damage (Figure S4D). Mitochondrial respiration was markedly impaired by PQ in control cells but maintained in AKAP1‐expressing RGCs (Figure S4E,F). In addition, AKAP1 reduced mitochondrial superoxide production as assessed by MitoSOX staining (Figure S4G,H). Three‐dimensional (3D) EM tomography analysis revealed that PQ induced mitochondrial fragmentation in control RGCs, characterized by reduced mitochondrial length and area. In contrast, AKAP1 preserved mitochondrial morphology, increased cristae density, and enhanced ATP production per mitochondrial volume (Figure S5A–C). These results indicate that AKAP1 mitigates oxidative injury by preserving mitochondrial structure and function.

### 2.8 AKAP1 is Associated With the Regulation of CaMKII and Synapsin Signaling

Given that synaptic dysfunction precedes RGC loss in glaucoma [12, 13, 14], we next examined whether AKAP1 influences synaptic signaling pathways. CaMKII is a central regulator of synaptic plasticity that becomes autonomously active upon autophosphorylation at Thr286 in response to intracellular calcium elevation, subsequently phosphorylating downstream synaptic substrates, including synapsins, to facilitate vesicle mobilization and neurotransmitter release [24, 25, 26, 27]. Phosphorylated CaMKII (pCaMKII) levels were significantly increased in D2‐Null retinas but normalized following AKAP1 restoration (Figure 5A–C). Similarly, phosphorylation of synapsin at Ser9, a downstream target of CaMKII [24], was elevated in glaucomatous retinas and reduced to baseline levels in D2‐AKAP1 retinas (Figure 5D–F). In contrast, total synapsin expression, which remained unchanged in D2‐Null retinas, was reduced after AKAP1 treatment in Western blot analysis, whereas immunohistochemical analysis showed no significant change in synapsin immunoreactivity (Figure S6A–C). These findings indicate that AKAP1 expression in the retina is associated with normalization of CaMKII and synapsin phosphorylation measured in whole‐retina lysates and inner retinal sections. They do not establish that AKAP1 acts directly on this pathway or that CaMKII‐synapsin signaling mediates the observed synaptic preservation.

**FIGURE 5:**
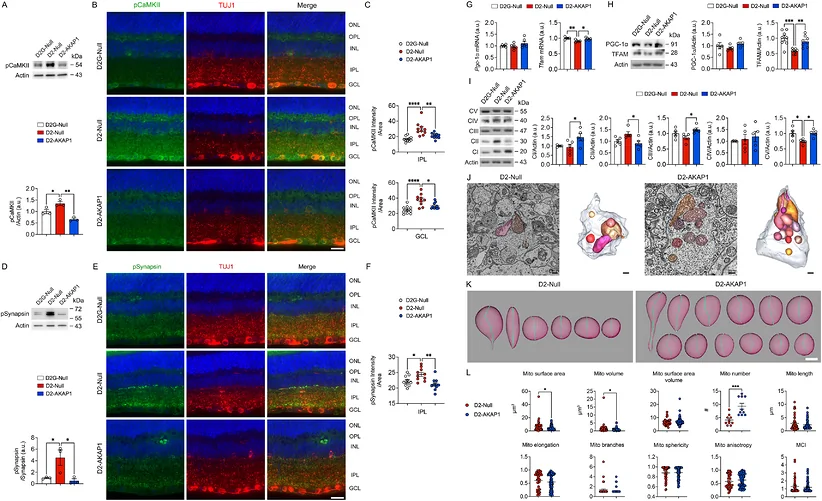
AKAP1 preserves synaptic activity and enhances mitochondrial biogenesis in the presynaptic terminals of glaucomatous D2 retinas. (A) Representative image of a Western blot and densitometry graph for pCaMKII expression in the retinas of D2G‐Null, D2‐Null, and D2‐AKAP1 mice (*n* = 3 retinas from 3 mice per group). (B) Representative retinal images for pCaMKII (green) and TUJ1 (red) immunoreactivities. (C) Quantitative fluorescent intensity of pCaMKII immunoreactivity in the IPL and GCL of the retinas (*n* = 10 retina sections from 3 mice per group). (D) Representative image of a Western blot and densitometry graph for pSynapsin (Ser9) expression in the retinas (*n* = 3 retinas from 3 mice per group). (E) Representative retinal images for pSynapsin (Ser9) (green) and TUJ1 (red) immunoreactivities. (F) Quantitative fluorescent intensity of pSynapsin (Ser9) immunoreactivity in the IPL of the retinas (*n* = 10 retina sections from 3 mice per group). (G) Quantitative PCR for *Pgc‐1α* and *Tfam* gene expression in the retinas (*n* = 5 to 6 retinas from 5 to 6 mice per group). (H) Representative image of a Western blot and densitometry graph for PGC‐1α and TFAM in the retinas (*n* = 5 to 8 retinas from 5 to 8 mice per group). (I) Representative image of a Western blot and densitometry graph for OXPHOS complex in the retinas of D2G‐Null, D2‐Null, and D2‐AKAP1 mice (*n* = 5 retinas from 5 mice per group). (J) Representative SBEM volume showing typical synaptic button structures in D2‐Null and D2‐AKAP1 retinas; mitochondria (several different colors) highlighted. (K) Expedited and accurate segmentation and analysis of mitochondria. Surface rendering showing round forms of mitochondria. (L) Measurements for mitochondrial surface area (*n* = 20 mitochondria from 2 mice per group), volume (*n* = 20 mitochondria from 2 mice per group), surface area volume (*n* = 20 mitochondria from 2 mice per group), number (*n* = 10 mitochondria from 2 mice per group), length (*n* = 20 mitochondria from 2 mice per group), elongation (*n* = 20 mitochondria from 2 mice per group), branches (*n* = 10 mitochondria from 2 mice per group), sphericity (*n* = 20 mitochondria from 2 mice per group), anisotropy (*n* = 20 mitochondria from 2 mice per group), and MCI (*n* = 20 mitochondria from 2 mice per group) in D2‐Null and D2‐AKAP1 buttons. Bars represent SEM. Statistical analysis was performed using unpaired Student's *t*‐test or one‐way ANOVA and Tukey's multiple comparisons test. ^*^ *p* < 0.05, ^**^ *p* < 0.01, ^***^ *p* < 0.001, and ^****^ *p* < 0.0001. Scale bars, 20 µm (*B* and *E*) and 500 nm (J, K).

### 2.9 AKAP1 Promotes Mitochondrial Biogenesis in Presynaptic Terminals

To investigate whether AKAP1 influences mitochondrial biogenesis, we assessed the expression of key regulators. AKAP1 restoration significantly increased mitochondrial transcription factor A (TFAM) expression at both mRNA and protein levels, whereas peroxisome proliferator‐activated receptor gamma coactivator 1‐alpha (PGC1‐α) expression remained unchanged (Figure 5G,H). In addition, expression of OXPHOS complexes was enhanced, indicating improved mitochondrial respiratory capacity (Figure 5I). Serial block‐face scanning electron microscopy (SBEM) analysis combined with 3D reconstruction revealed that AKAP1 restoration increased mitochondrial number within presynaptic terminals (Figure 5J–L; see also Movies S4,S5). However, mitochondrial morphology parameters, including length, branching, sphericity, anisotropy, and mitochondrial complexity index (MCI) [28, 29], remained largely unchanged. These findings suggest that retinal AKAP1 expression increases mitochondrial content within IPL presynaptic terminals. Because TFAM, PGC1‐α, and OXPHOS complexes were measured in whole‐retina lysates, the cellular origin of these biochemical changes cannot be assigned to RGCs specifically.

### 2.10 AKAP1 Enhances Ribbon Synapse Integrity and Vesicle Organization

Using SBEM and 3D reconstruction, we examined ribbon synapse ultrastructure in IPL synaptic terminals of D2‐Null and D2‐AKAP1 retinas [30, 31, 32]. Representative SBEM volumes revealed that D2‐Null terminals contained six ribbon synapses and a single elongated spinule with limited presynaptic invasion, whereas D2‐AKAP1 terminals contained nine ribbon synapses and multiple bulbous spinules that penetrated deeply into the presynaptic cytoplasm and were densely filled with vesicles (Figure 6A,B; see also Movies S6–S9). Quantitative analysis confirmed that AKAP1 restoration significantly increased both ribbon synapse and spinule number per terminal, while ribbon length and surface area per volume were comparable between groups (Figure 6C). Given that synaptic mitochondria are critical for local energy supply and calcium buffering [33, 34, 35], we quantified the spatial proximity of mitochondria to ribbon synapses within presynaptic terminals (Figure 6D). D2‐AKAP1 retinas showed a significant increase in mitochondria positioned within defined proximity ranges of ribbon synapses relative to D2‐Null retinas (Figure 6E), indicating improved mitochondrial positioning at active zones. We further quantified the distance between the synaptic ribbon and its rapidly releasable vesicle pool, a spatial parameter linked to synaptic transmission efficacy and neurodegeneration [36, 37, 38], and found a significantly reduced ribbon‐to‐vesicle pool distance in D2‐AKAP1 retinas compared to D2‐Null retinas (Figure 6F–I). To quantify total synaptic vesicle number, a direct determinant of neurotransmitter release capacity, we applied SBEM in combination with the machine‐learning segmentation platform CDeep3M [39, 40, 41, 42], enabling high‐throughput, unbiased volumetric analysis of presynaptic terminals (Figure 7A–D). D2‐AKAP1 retinas exhibited significantly greater total vesicle counts per terminal than D2‐Null retinas (Figure 7E,F; see also Movies S10,S11). Collectively, these structural changes indicate that AKAP1 improves synaptic connectivity and neurotransmission capacity in the inner retinal synaptic architecture.

**FIGURE 6:**
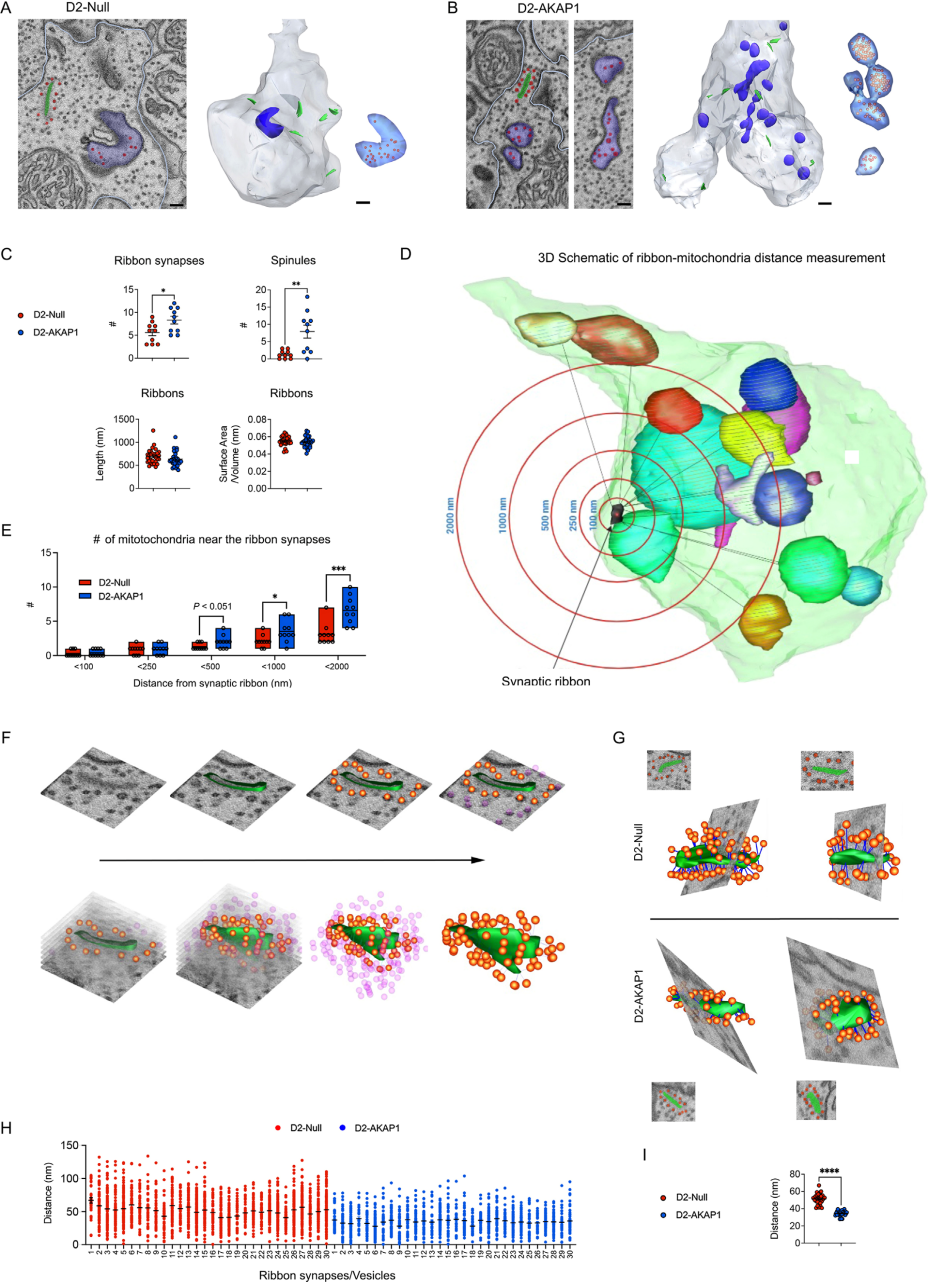
AKAP1 enhances ribbon synapse integrity and mitochondrial proximity in glaucomatous D2 retinas. (A, B) Representative SBEM volume showing typical synaptic bouton structures in D2‐Null and D2‐AKAP1 retinas; ribbon synapses (green), synaptic vesicles (red), and spinules (blue) are highlighted. (C) Measurements for the number of ribbon synapses and spinules (*n* = 10 synaptic boutons from 2 mice per group), and synaptic ribbon length and synaptic ribbon surface area per volume (*n* = 30 synaptic ribbons from 2 mice per group). (D) 3D schematic for measurements of ribbon‐mitochondria distance. (E) Measurements for the number of mitochondria near ribbon synapses within a defined distance (*n* = 10 synaptic boutons from 2 mice per group). (F) Representative SBEM images for synaptic bouton (gray), synaptic ribbon (green), and synaptic vesicles (orange dots) of D2‐Null and D2‐AKAP1 retina. (G) Representative images for SBEM and 3D IMOD analysis showing typical synaptic ribbons and vesicles in D2‐Null and D2‐AKAP1 retinas; synaptic ribbons (green) and synaptic vesicles (red) are highlighted. (H) Total measurements for the distance between ribbon synapses and vesicles in D2‐Null and D2‐AKAP1 retinas (*n* = 30 synaptic ribbons from 2 mice per group). (I) Averaged measurement for the distance between ribbon synapses and vesicles in D2‐Null and D2‐AKAP1 retinas (*n* = 30 synaptic ribbons from 2 mice per group). SBEM sampling, segmentation, and quantification were blinded. Error bars represent SEM. Statistical analysis was performed using an unpaired Student's *t*‐test. ^*^ *p* < 0.05, ^**^ *p* < 0.01, ^***^ *p* < 0.001, and ^****^ *p* < 0.0001. Scale bars, 500 nm (A, B).

**FIGURE 7:**
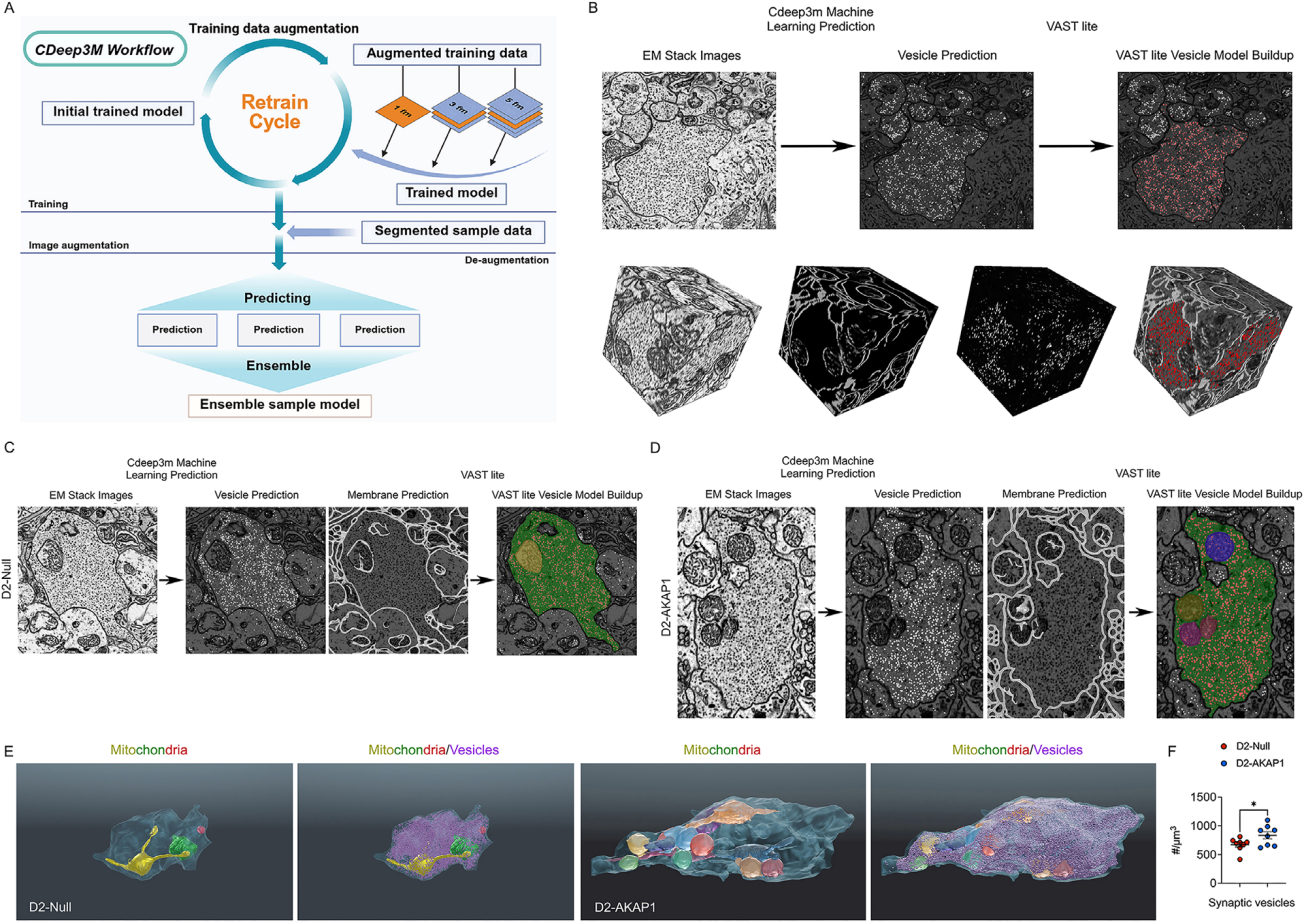
AKAP1 promotes vesicle pools in glaucomatous D2 retinas. (A) Schematic summary of the AI‐based segmentation tool CDeep3M workflow to facilitate deep learning‐based image segmentation of 3D image stacks. (B) Top row: A representative 2D EM image from a stack (left) is used as input to a machine‐learning‐based segmentation using CDeep3M. The pipeline predicts synaptic vesicles (middle), which are then used as input to build the model in VAST Lite (right). The overlay in the final image shows vesicles segmented in red. Bottom row: 3D renderings illustrate the pipeline progression. From left to right: raw EM image volume, membrane prediction, vesicle prediction, and automated vesicle segmentation. Red spheres denote segmented synaptic vesicles within the synaptic boutons. (C) D2‐Null. 3D renderings illustrate the pipeline progression. From left to right: EM stack images, vesicle prediction, membrane prediction, and VAST Lite vesicle model buildup. The overlay in the final image shows vesicles segmented in red and mitochondria in several colors. (D) D2‐AKAP1. 3D renderings illustrate the pipeline progression. From left to right: EM stack images, vesicle prediction, membrane prediction, and VAST Lite vesicle model buildup. The overlay in the final image shows vesicles segmented in red and mitochondria in several colors. (E) Representative images for volume‐rendered 3D models of mitochondria and synaptic vesicles using CDeep3M and Vast Lite. (F) Measurements for the number of synaptic vesicles using CDeep3M and Vast Lite (*n* = 8 synaptic boutons from 2 mice per group). SBEM sampling, segmentation, and quantification were blinded. Error bars represent SEM. Statistical analysis was performed using an unpaired Student's *t*‐test. ^*^ *p* < 0.05. Panel (A) includes graphical elements created by BioRender (www.biorender.com).

### 2.11 AKAP1 Preserves Synaptic Integrity and Suppresses Neuroinflammation

We examined whether AKAP1 modulates synaptic integrity and neuroinflammatory responses. Synaptophysin expression, which was reduced in glaucomatous retinas, was improved by AKAP1 expression (Figure 8A–C). In contrast, complement component C1q, a marker of synapse elimination [12, 43], was elevated in D2‐Null retinas and reduced following AKAP1 restoration (Figure 8D,E). Markers of glial activation, including IBA1 and GFAP, were significantly increased in glaucomatous retinas but attenuated by AKAP1 expression (Figure 8F–J). In addition, expression of inflammatory mediators, including *Nlrp3*, *Caspase‐1*, *Il‐1β*, and *Tnf‐α*, was significantly reduced following AKAP1 restoration (Figure 8K). AKAP1 also improved PSD95 expression (Figure S7A–C), indicating preservation of postsynaptic structure. Collectively, these findings demonstrate that retinal AKAP1 expression is associated with preserved synaptic integrity, reduced complement and neuroinflammatory markers, and maintenance of both pre‐ and postsynaptic architecture. Because these measurements were obtained from whole‐retina lysates and inner retinal sections in eyes with less overall damage, the lower C1q, IBA1, and GFAP levels may reflect reduced tissue injury rather than a direct anti‐complement or anti‐inflammatory action of AKAP1.

**FIGURE 8:**
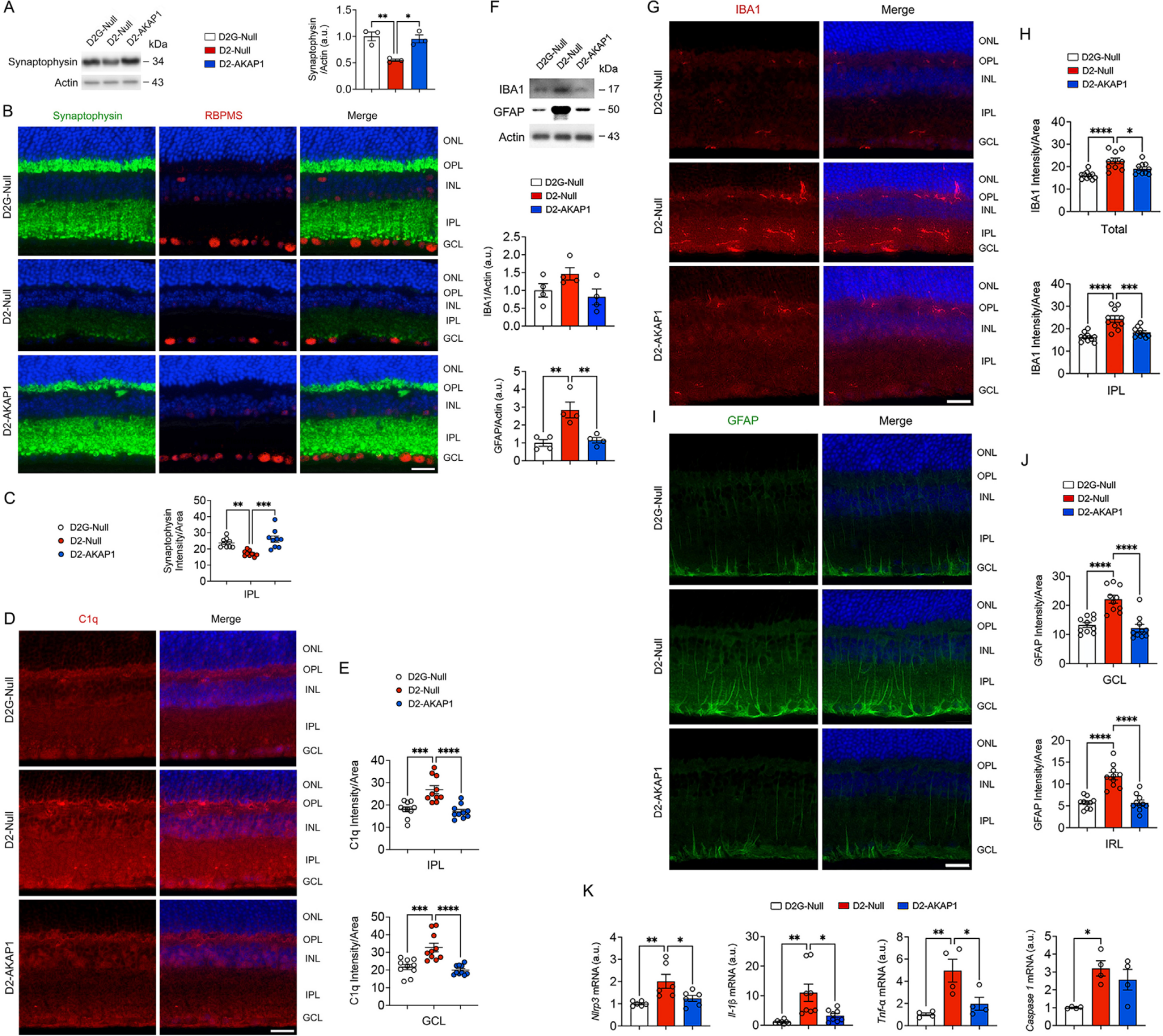
AKAP1 preserved synaptic activity by increasing synaptophysin but decreasing C1q expression in glaucomatous D2 retinas. (A) Representative image of a Western blot and densitometry graph for synaptophysin expression in the retinas of D2G‐Null, D2‐Null, and D2‐AKAP1 mice (*n* = 3 retinas from 3 mice per group). (B) Representative retinal images for synaptophysin (green) and RBPMS (red) immunoreactivities. (C) Quantitative fluorescent intensity of synaptophysin immunoreactivity in the IPL of the retinas (*n* = 8 retinal sections from 3 mice per group). (D) Representative retinal images for C1q (green) immunoreactivity. (E) Quantitative fluorescent intensity of C1q immunoreactivity in the IPL and GCL of the retinas (*n* = 10 retina sections from 3 mice per group). (F) Western blot analysis for IBA1 and GFAP expression in glaucomatous D2 retinas (*n* = 4 retinas from 4 mice per group). (G) Representative retinal images for IBA1 (red) immunoreactivity in glaucomatous D2 retinas. (H) Quantitative fluorescent intensity of IBA1 immunoreactivity in the IPL and total layer that includes OPL, ONL, INL, IPL, and GCL of the normal and glaucomatous D2 retinas (*n* = 10 retinal sections from 3 mice per group). (I) Representative retinal images for GFAP (green) immunoreactivity in glaucomatous D2 retinas. (J) Quantitative fluorescent intensity of GFAP immunoreactivity in the GCL and inner retinal layer (IRL: IRL includes INL, IPL, and GCL) in glaucomatous D2 retinas (*n* = 10 retinal sections from 3 mice per group). (K) Quantitative PCR analysis for *Nlrp3*, *Il‐1β*, *Tnf‐α*, and *Caspase1* gene expression in glaucomatous D2 retinas (*n* = 4 to 6 retinas from 4 to 6 mice per group). Error bars represent SEM. Statistical analysis was performed using one‐way ANOVA and Tukey's multiple comparisons test. *p* < 0.05, ^**^ *p* < 0.01, ^***^ *p* < 0.001, and ^****^ *p* < 0.0001. Scale bars, 20 µm.

## 3 Discussion

Glaucoma affects more than 80 million people worldwide, yet current therapies are largely limited to lowering IOP, which slows but does not prevent disease progression. This limitation reflects a fundamental gap in our ability to directly target the cellular mechanisms underlying RGC degeneration [44]. Among these, mitochondrial dysfunction has emerged as a central pathogenic feature, contributing to impaired bioenergetics, increased oxidative stress, and activation of apoptotic pathways in vulnerable neuronal compartments [2, 3, 6, 44, 45, 46]. However, translating this knowledge into effective neuroprotective strategies has been challenging, in part due to an incomplete understanding of how mitochondrial dysregulation integrates with synaptic failure and neuroinflammation in glaucoma.

In this study, we identify AKAP1 as a critical regulator of this interface and demonstrate that its restoration provides robust neuroprotection across two distinct glaucoma models. AAV2‐mediated AKAP1 delivery preserved RGC survival, maintained visual function, and improved retina‐to‐brain connectivity despite sustained IOP elevation, indicating that its protective effects act downstream of pressure‐induced stress. Importantly, the consistency of these outcomes in both the DBA/2J and microbead‐induced ocular hypertension models strengthens the generalizability of AKAP1‐mediated neuroprotection and reduces the likelihood that these effects are model‐specific. Conversely, AKAP1 deficiency selectively exacerbated functional impairment without significantly accelerating RGC loss, suggesting that AKAP1 is particularly important for maintaining neuronal performance under stress conditions. We note, however, that AAV‐mediated AKAP1 expression was not restricted to RGCs; we therefore attribute the observed neuroprotection to AKAP1 restoration in the retina more broadly and cannot exclude a contribution from non‐cell‐autonomous mechanisms. Together, these findings position AKAP1 not only as a marker of mitochondrial health but as an active determinant of inner retinal neuron resilience, including RGCs. Future studies will employ cell‐type‐specific expression strategies to define the precise cellular mechanism in glaucomatous neurodegeneration.

A central insight of this study is that retinal AKAP1 expression is associated with the regulation of mitochondrial dynamics and bioenergetic capacity in the glaucomatous retina. Rather than broadly reiterating mitochondrial dysfunction as a feature of disease, our data specify how AKAP1 regulates this process by modulating the CaN–DRP1 axis and promoting mitochondrial fusion. By increasing DRP1 phosphorylation at S637 and increasing expression of the fusion protein OPA1, particularly the fusion‐competent long form, without detectable changes in MFN1 or MFN2, AKAP1 shifts the balance toward a fused mitochondrial network that is structurally and functionally optimized for energy production. This is supported by ultrastructural evidence demonstrating increased cristae density and mitochondrial volume fraction, as well as functional measurements showing enhanced oxidative phosphorylation and ATP production. These findings extend prior work demonstrating that AKAP1 inhibits DRP1‐mediated mitochondrial fission [10, 11] by establishing that this mechanism operates in vivo during glaucomatous neurodegeneration and is associated with improved mitochondrial structure and bioenergetic capacity under chronic glaucomatous stress.

Beyond mitochondrial structure, our results highlight a critical role for AKAP1 in linking mitochondrial function to synaptic integrity. Synaptic dysfunction is increasingly recognized as an early event in glaucoma progression, often preceding overt RGC loss [14, 15, 16, 47, 48, 49]. However, the mechanistic link between mitochondrial impairment and synaptic failure remains poorly defined. Here, we demonstrate that AKAP1 restoration enhances mitochondrial biogenesis within presynaptic terminals, increases mitochondrial availability at synaptic sites, and improves mitochondrial positioning relative to ribbon synapses. These changes are accompanied by increased synaptic vesicle number, reduced vesicle‐ribbon distance, and improved ribbon synapse structure, indicating that mitochondrial support is directly coupled to synaptic architecture and function. Given the high energetic demands of synaptic transmission [48, 50, 51], these findings support a model in which AKAP1 maintains synaptic competence by ensuring an adequate local energy supply.

At the signaling level, our data further reveal that AKAP1 modulates key pathways controlling synaptic activity, particularly the CaMKII–synapsin axis. Dysregulated calcium signaling and aberrant CaMKII activation have been implicated in glaucomatous neurodegeneration [22, 52, 53], yet their relationship to mitochondrial dysfunction has remained unclear. AKAP1 normalized CaMKII and synapsin phosphorylation toward control levels. Because both are sensitive to Ca^2^^+^ and cellular energy status, these changes are consistent with improved mitochondrial homeostasis and reduced cellular stress in the inner retina, although we cannot exclude that they reflect parallel rather than downstream effects of AKAP1. This interpretation is consistent with the established role of mitochondria in calcium homeostasis and with prior evidence that CaMKII activity is sensitive to metabolic stress and rescues RGCs [22, 52, 53]. Normalization of synapsin phosphorylation may contribute to improved vesicle clustering and neurotransmitter release, given that synapsins are key regulators of vesicle pool organization [26, 54, 55, 56, 57, 58]. In parallel, restoration of synaptophysin expression further supports the preservation of presynaptic structure and function. Together, these findings are consistent with, but do not establish, a signaling axis through which AKAP1 couples mitochondrial health to synaptic transmission; targeted pathway‐intervention experiments will be required to test this directly. As we and others have reported, the DBA/2J model exhibits marked heterogeneity of glaucomatous injury at 10 months of age, with individual eyes spanning no‐or‐early, moderate, and severe damage stages on optic nerve grading [59, 60, 61]. We acknowledge as a limitation of the present study that per‐sample optic nerve grading was not available for the EM cohort; consequently, unmeasured variability in disease stage may contribute to the variance in our ultrastructural metrics. Future work will directly correlate ultrastructural measures with optic nerve damage grade in larger, stratified cohorts.

In addition to presynaptic effects, AKAP1 also appears to preserve postsynaptic integrity. Restoration of PSD95 expression in glaucomatous retinas indicates stabilization of postsynaptic density architecture, which is known to be disrupted early in disease progression [62, 63]. Because PSD95 is regulated in part by CaMKII‐dependent signaling, its preservation further supports the idea that AKAP1‐mediated control of calcium and kinase activity extends across synaptic compartments. This coordinated preservation of pre‐ and postsynaptic elements suggests that AKAP1 functions as a broader regulator of synaptic homeostasis, rather than acting solely through mitochondrial bioenergetics.

Another important contribution of this study is the identification of a link between AKAP1 and neuroinflammatory processes. Complement‐mediated synapse elimination has been implicated as an early driver of synaptic loss in glaucoma [12, 43, 64, 65], yet strategies to modulate this pathway remain limited. We show that retinal AKAP1 expression is associated with lower C1q expression, reduced glial activation, and lower levels of inflammasome‐associated cytokine transcripts. These findings suggest that AKAP1 not only preserves neuronal intrinsic function but also modulates the extrinsic inflammatory environment that contributes to neurodegeneration. An equally plausible interpretation, which our data cannot exclude, is that these differences are secondary to reduced overall tissue injury in AKAP1‐treated eyes rather than a direct anti‐complement or anti‐inflammatory action of AKAP1. One plausible mechanism is that improved mitochondrial integrity reduces the release of damage‐associated molecular patterns that activate complement and glial responses, although this will require direct investigation. Regardless of the precise mechanism, the association between retinal AKAP1 expression and lower levels of multiple neuroinflammatory markers highlights its potential as a multifunctional therapeutic target.

A notable aspect of our findings is the dissociation between structural and functional outcomes in AKAP1*^−/−^* mice. Although RGC numbers were not significantly further reduced in AKAP1*^−/−^* mice under ocular hypertension, functional deficits were exacerbated, as reflected by decreased pERG amplitudes. This observation reinforces the concept that neuronal dysfunction precedes cell death in glaucoma and suggests that AKAP1 plays a particularly important role in maintaining physiological performance under stress. Importantly, the apparently preserved structural phenotype may reflect developmental compensatory mechanisms associated with germline AKAP1 deletion, including compensation by other AKAP family members or alternative regulators of mitochondrial dynamics. However, we also cannot exclude that our structural analysis was insufficiently powered to detect a modest difference in RGC number. Thus, inducible or RGC‐specific AKAP1 knockout models will be needed to distinguish compensatory effects from a direct role for AKAP1 in glaucomatous neurodegeneration. Together with our AKAP1 restoration data, these findings suggest that AKAP1‐dependent signaling supports RGC function under glaucomatous stress and identify it as a candidate target for functional preservation, a possibility that will require direct testing in interventional studies.

The translational implications of these findings are significant. AAV2‐mediated gene delivery has already demonstrated safety and efficacy in clinical settings for retinal diseases, providing a feasible platform for AKAP1‐based therapies. Importantly, the neuroprotective effects observed here occur independently of IOP reduction, suggesting that AKAP1 therapy could complement rather than replace existing treatments. This raises the possibility of combination strategies that simultaneously address upstream risk factors and downstream neurodegenerative mechanisms. Several limitations should be considered. First, although AKAP1 deficiency was accompanied by lower PKA protein in the retina and AAV2‐AKAP1 increased retinal PKA levels, we did not measure PKA catalytic activity, mitochondrial PKA recruitment, or AKAP1‐PKA complex formation. The AKAP1‐PKA axis therefore remains a plausible but mechanistically unproven component of the model presented here. Second, the CMV‐driven AAV2 construct was not restricted to RGCs, and most biochemical and ultrastructural measurements were obtained from whole‐retina lysates, the inner retina, or the IPL; our findings therefore describe the consequences of retinal AKAP1 restoration rather than an RGC‐autonomous effect, and this applies in particular to the mitochondrial, CaMKII/synapsin, and complement data. Third, IOP after microbead injection was modestly but significantly higher in AKAP1‐deficient mice, so a contribution of pressure to the exacerbated pERG deficit cannot be excluded. Fourth, purity of the immunopanned RGC cultures was not quantified in the present experiments and was inferred from prior studies using the same protocol. Finally, the precise molecular interactions linking AKAP1 to calcium signaling and complement activation remain to be defined, and the CaMKII, synapsin, and complement findings remain associative in the absence of pathway‐intervention experiments. In addition, while two experimental models were used, further validation in large‐animal models and human tissue will be important for clinical translation. Finally, long‐term studies will be necessary to determine the durability of AAV2‐AKAP1–mediated protection and its potential effects on disease progression over extended timeframes.

In conclusion, this study identifies AKAP1 as a candidate regulator of mitochondrial and synaptic homeostasis in glaucomatous retina (Figure S8) and demonstrates that its restoration provides robust neuroprotection across multiple levels of disease pathology. Retinal AKAP1 expression was accompanied by enhanced mitochondrial fusion and mitochondrial content, improved bioenergetic capacity, preserved synaptic structure, and lower neuroinflammatory markers, each of which has been implicated in RGC degeneration. These findings support a model in which mitochondrial dysfunction, synaptic failure, and inflammation are tightly interconnected processes that can be coordinately targeted through AKAP1 signaling. As such, AKAP1 represents a promising therapeutic target for preserving vision in glaucoma and, potentially, in other neurodegenerative diseases characterized by mitochondrial and synaptic dysfunction.

## 4 Methods

### 4.1 Animals

Adult male and female D2 and age‐matched D2G control mice, C57BL/6J mice (all from The Jackson Laboratory), and AKAP1*^−/−^* mice were housed under standard conditions (covered cages, ad libitum standard rodent diet, 12 h light/dark cycle). AKAP1*^−/^* ^+^ mice, generously provided by Dr. Stefan Strack (University of Iowa), were intercrossed to generate AKAP1*^−/−^* offspring [10]. Genotyping was performed by PCR using the following primers: GGAGGCGATCACAGCAACAACCG (R), ATACAGAAGCAGATCACTCAGGAGG (F‐WT), and CAGTCCCAAGGCTCATTTCAGGCC (F‐KO), as previously described [10, 11]. For AAV2‐AKAP1 experiments, 5‐month‐old D2 and age‐matched D2G mice, and 3‐month‐old C57BL/6J and AKAP1*^−/−^* mice were used. Animals were randomly assigned to experimental and control groups. All procedures conformed to the ARVO Statement for the Use of Animals in Ophthalmic and Vision Research and were approved by the Institutional Animal Care and Use Committee at the University of California, San Diego.

### 4.2 Production of Recombinant AAV2‐AKAP1 Vector

The mouse Akap1 gene (nucleotides 1–2571) was cloned into the pAAV‐MCS vector (Agilent Technologies). The AAV2‐AKAP1 expression cassette consisted of the CMV promoter, the Akap1 coding sequence fused to a C‐terminal 6x‐His tag only, and the human growth hormone (hGH) poly A signal (nucleotides 1–478) (Figure S1C). An AAV‐Null vector was used as a control. Recombinant AAV2‐AKAP1‐His and the AAV2‐Null vectors were produced by co‐transfecting AAV‐293 cells (Agilent Technologies) with the AAV2‐AKAP1‐His or AAV2‐Null transfer plasmid, the pHelper plasmid (Agilent Technologies), and the pM4‐Rep/Cap plasmid [66]. Viral particles were purified according to a previously published protocol [67], and viral titers were determined by qPCR using primers targeting the inverted terminal repeats (ITRs; TaKaRa Bio Inc).

### 4.3 Transduction With Recombinant AAV2 Constructs In Vivo and In Vitro

For intravitreal injections, mice were anesthetized with an intraperitoneal (IP) injection of ketamine (100 mg/kg, Ketaset; Fort Dodge Animal Health) and xylazine (9 mg/kg, TranquiVed; VEDCO Inc.), followed by topical application of 1% proparacaine hydrochloride for corneal anesthesia. The globe was punctured using the bevel of a 30‐gauge needle, and a 33‐gauge Hamilton syringe was inserted through the puncture site. A total of 1 µL of either AAV2‐Null or AAV2‐AKAP1 (1 × 10^1^ ^3^ genome copies/mL [gc/mL]) was slowly injected into the vitreous humor over 1 min, and the needle was held in place for an additional 5 min to minimize vector reflux through the injection tract [39, 45]. For in vitro transduction, primary RGCs were treated with either AAV2‐Null or AAV2‐AKAP1 for 3 days prior to experimental manipulation.

### 4.4 Mouse Model of MB‐Induced OHT

OHT was induced using a modified MB occlusion protocol, as previously described [39]. In brief, 6‐week‐old C57BL/6J mice were intravitreally injected with either AAV2‐Null or AAV2‐AKAP1 as described above. At 3 weeks post‐injection, mice were anesthetized by an IP injection of ketamine/xylazine. We topically applied 0.5% proparacaine hydrochloride, dilated the pupils with a 1:1 mixture of 1% tropicamide and 2.5% phenylephrine, and then gently punctured the cornea with a 30‐gauge needle. Next, 2 µL of 1‐µm‐diameter polystyrene MB suspension (containing 3.0 × 10^7^ beads) followed by an air bubble, 2 µL of 6‐µm‐diameter polystyrene MB suspension (containing 6.3 × 10^6^ beads, Polysciences), and 1 µL of PBS containing 30% Healon was injected into the anterior chamber using a 32‐gauge needle (Hamilton Company) connected to a syringe (Hamilton Company), which induced moderate elevation of IOP for ≥ 8 weeks. An equivalent volume of PBS was injected into the contralateral eyes to serve as a sham control.

IOP was measured weekly or biweekly for 8 weeks using a tonometer. At 8 weeks after MB injection, mice were tested for visual function, then sacrificed, and their retinas were isolated.

### 4.5 Primary RGCs Isolation and Purification

Primary RGCs were isolated from postnatal day 3–5 Sprague‐Dawley rats by immunopanning and maintained in serum‐free, defined growth medium, as previously described [45, 68, 69]. Briefly, culture vessels were sequentially coated with poly‐D‐lysine (70 kDa, 10 µg/mL; Sigma–Aldrich) followed by laminin (10 µg/mL; Sigma–Aldrich) in Neurobasal medium. Purified RGCs were seeded at the following densities: 2 × 10^5^ cells per 60 mm dish, 1 × 10^5^ cells per well in 24‐well plates, and 1.5 × 10^4^ cells per well in 96‐well plates. Growth medium was supplemented with brain‐derived neurotrophic factor (BDNF; 50 ng/mL; Sigma–Aldrich), ciliary neurotrophic factor (CNTF; 10 ng/mL; Sigma–Aldrich), insulin (5 µg/mL; Sigma–Aldrich), and forskolin (10 µg/mL; Sigma–Aldrich). Culture purity was not quantified in the present study and is inferred from prior work using this immunopanning protocol; the absence of experiment‐specific quantification of RBPMS‐ or BRN3A‐positive cells is acknowledged as a limitation.

### 4.6 Elevated HP and PQ Treatment in Primary RGCs In Vitro

To model mechanical and oxidative stress in vitro, cultured RGCs were subjected to either elevated HP or PQ (Sigma–Aldrich) treatment 3 days post‐transduction with AAV2‐AKAP1 or AAV2‐Null, as previously described [45, 69]. For elevated HP treatment, culture plates were placed in a sealed plexiglass pressure chamber connected to a certified 5% CO_2_/95% air gas source (Airgas Inc.) via a low‐pressure two‐stage regulator (Gilmont Instruments) and housed within a standard tissue culture incubator at 37°C with 5% CO_2_. NP and elevated HP conditions were maintained at 15 mmHg and 30 mmHg above atmospheric pressure, respectively, for 24 h. For oxidative stress treatment, RGCs were exposed to 50 µm PQ (Sigma–Aldrich) diluted in complete growth medium for 24 h. Mitochondrial ROS generation was assessed using MitoSOX Red staining (Invitrogen). Following treatment under either elevated HP or PQ, RGCs were subjected to downstream analyses, including cell viability (MTT assay), cytotoxicity (LDH assay), mitochondrial ROS detection, Western blot analysis of mitochondrial and apoptotic markers, and mitochondrial respiration profiling via OCR measurement.

### 4.7 IOP Measurement

IOP was measured using a rebound tonometer (Icare TONOVET) under light isoflurane anesthesia, as previously described [39, 45, 46]. Five consecutive measurements were obtained per eye and automatically averaged by the device. In D2 mice, IOP elevation typically begins between 5 and 7 months of age, with significant ON axon damage evident by 9–10 months [39, 45, 46]. The IOP was assessed as previously described, and 65.3% (64/98) of glaucomatous D2 mice assessed at 10 months exhibited confirmed IOP elevation, with a mean peak IOP of 21.5 ± 4.5 mmHg in the right eye and 19.9 ± 3.7 mmHg in the left eye. Only D2 mice with a spontaneous IOP increase exceeding 20 mmHg were included in the study. IOP was similarly measured in non‐glaucomatous D2G and C57BL/6J control mice (*n* = 5 measurements per eye).

### 4.8 Tissue Preparation

At the end of each experiment, mice were anesthetized with an IP injection of ketamine/xylazine as described above and euthanized by cervical dislocation. Eyes were enucleated and processed according to the intended downstream analysis. For immunohistochemical analysis, eyes were fixed in 4% paraformaldehyde (PFA) in phosphate‐buffered saline (PBS, pH 7.4; Sigma–Aldrich) for 4 h at 4°C, after which retinas were dissected and prepared as flattened whole‐mounts. For Western blot analysis and quantitative RT‐PCR (qRT‐PCR), retinas were immediately isolated and snap‐processed without fixation. For superior colliculus (SC) tissue, mice underwent cardiac perfusion with 0.9% saline followed by 4% PFA in PBS (pH 7.4; Sigma–Aldrich), and brain tissue was subsequently processed for cryosectioning. For EM, eyes were fixed by cardiac perfusion with 2% PFA and 2.5% glutaraldehyde in 0.15 m sodium cacodylate buffer (pH 7.4) at 37°C, followed by immersion in the same pre‐cooled fixative on ice for 1 h.

### 4.9 Western Blot Analysis

Western blot analysis was performed, as previously described [39, 45, 46]. Harvested retinas were homogenized on ice for 1 min in RIPA lysis buffer [25 mm Tris‐HCl (pH 7.6), 150 mm NaCl, 1% NP‐40, 0.5% deoxycholic acid, 0.1% sodium dodecyl sulfate (SDS); Thermo Scientific] supplemented with a protease inhibitor cocktail (Thermo Scientific). Lysates were centrifuged at 13 000 × g for 15 min at 4°C, and protein concentration in the clarified supernatant was determined using the BCA Protein Assay Kit (Thermo Scientific). Equal amounts of protein (10–20 µg) were resolved on 4%–20% Mini‐PROTEAN TGX precast gels (Bio‐Rad) and transferred to polyvinylidene difluoride (PVDF) membranes (GE Healthcare Biosciences). Briefly, membranes were blocked in Tris‐buffered saline with 0.1% Tween‐20 (TBST) containing 5% non‐fat dry milk for 1 h at room temperature, then incubated with primary antibodies (Table S4) overnight at 4°C. After three washes with TBST, membranes were incubated with horseradish peroxidase (HRP)‐conjugated secondary antibodies (Cell Signaling Technology) for 1 h at room temperature. Chemiluminescent signal was detected using an enhanced chemiluminescence (ECL) substrate (Thermo Fisher Scientific) and imaged with a ChemiDoc Imaging System (Bio‐Rad).

### 4.10 Immunohistochemistry

Immunohistochemical staining was performed on 7 µm paraffin‐embedded sections of full‐thickness retina. To block non‐specific binding, sections were incubated in 1% bovine serum albumin (BSA) in PBS for 1 h at room temperature, followed by overnight incubation with primary antibodies (Table S4) at 4°C. After three washes in PBS, sections were incubated with species‐appropriate fluorescently conjugated secondary antibodies for 4 h at 4°C. Nuclei were counterstained with Hoechst 33342 (1 µg/mL in PBS). Fluorescence images were acquired using a Keyence BZ‐X810 All‐in‐One Fluorescence Microscope (Keyence Corp. of America) with identical acquisition parameters across all groups. Integrated fluorescence intensity per unit area was quantified in ImageJ (National Institutes of Health) with uniform background subtraction applied to all images.

### 4.11 Whole‐Mount Immunohistochemistry and RGC Counting

Retinas from enucleated eyes were dissected and prepared as flattened whole‐mounts. For cryoprotection, retinas were immersed in 30% sucrose in PBS for 24 h at 4°C. Non‐specific binding was blocked by incubation in a solution containing 3% donkey serum, 1% BSA, 1% fish gelatin, and 0.1% Triton X‐100 for 1 h at room temperature. Retinas were then incubated with primary antibodies, rabbit polyclonal anti‐RBPMS or mouse monoclonal anti‐TUJ1 (Table S4), for 3 days at 4°C. After three washes in PBS, retinas were incubated for 24 h at 4°C with Alexa Fluor 488‐conjugated donkey anti‐rabbit IgG or Alexa Fluor 568‐conjugated donkey anti‐mouse IgG secondary antibodies, followed by three additional PBS washes. Fluorescence images were acquired using a Keyence BZ‐X810 All‐in‐One Fluorescence Microscope (Keyence Corp. of America) using identical acquisition settings (objective, exposure time, gain, and illumination intensity) for all groups within an experiment; all images within a given comparison were processed identically. RBPMS‐ or TUJ1‐positive RGCs were counted in two standardized retinal zones corresponding to the middle (three‐sixths of the retinal radius) and peripheral (five‐sixths of the retinal radius) retina, and counts were averaged across zones using ImageJ (National Institutes of Health).

### 4.12 pERG and pVEP Analyses

pERG and pVEP responses were recorded using the Celeris system (Diagnosys LLC) according to the manufacturer's specifications, as previously described [39, 70]. For pERG recordings, alternating black‐and‐white vertical gratings were presented at 1 Hz (2 reversals per second) at a luminance of 50 cd/m^2^ via the corneal pattern stimulator. A total of 200 traces were recorded per eye and averaged, with pERG amplitude measured from the P1 peak to the N2 trough (µV). For pVEP recordings, each eye was stimulated with 100 white light flashes at 1 Hz and 0.05 cd·s/m^2^ via corneal stimulators, with responses recorded over a 300 ms window at a sampling frequency of 2000 Hz. A minimum of 3 trials per mouse were performed, with 200 traces averaged per eye across 100 sweeps per trial. pVEP amplitude was measured from the P1 peak to the N1 trough (µV), and latency was also recorded. Bandpass filter cutoffs were set at 1.25 Hz (low) and 100 Hz (high). All data were analyzed using Espion V6 software (Diagnosys LLC).

### 4.13 CTB Labeling

Anterograde labeling of retinofugal projections to the SC was performed using CTB, as previously described [39]. Using a Hamilton syringe, 1 µL of Alexa Fluor 594‐conjugated CTB (Thermo Fisher Scientific) was slowly injected into the vitreous humor over 1 min, and the needle was held in place for an additional 5 min to minimize tracer reflux through the injection tract. Three days post‐injection, mice were deeply anesthetized with an IP injection of ketamine/xylazine and transcardially perfused with 4% paraformaldehyde (PFA; Ted Pella) in PBS. Dissected SC tissues were immersed in PBS containing 30% sucrose for 24 h at 4°C for cryoprotection. Fluorescence images were acquired using a Keyence BZ‐X810 All‐in‐One Fluorescence Microscope (Keyence Corp. of America). CTB‐labeled area density in the SC was quantified using ImageJ (National Institutes of Health).

### 4.14 Transmission EM Analysis

Primary RGCs were cultured on poly‐D‐lysine‐coated MatTek dishes and processed for transmission EM, as previously described [39, 70]. For transmission EM analysis, the growth medium from cell culture dishes was removed, and cells were immediately fixed with a primary fixative containing 2% paraformaldehyde and 2.5% glutaraldehyde (Ted Pella) in 0.15 m sodium cacodylate buffer (pH 7.4) at 37°C for 2 min, then gradually cooled on ice for 20 min. Cells were washed three times (3 min each) in ice‐cold sodium cacodylate buffer and post‐fixed in a mixture of 1% osmium tetroxide (OsO_4_), 0.8% potassium ferrocyanide, and 3 µm calcium chloride in sodium cacodylate buffer for 30 min on ice. Following three washes in ice‐cold double‐distilled water (3 min each), cells were stained with 2% uranyl acetate for 30 min on ice. Cells were dehydrated through a graded ethanol series (20%, 50%, 70%, and 90% on ice, then 3% × 100% at room temperature, 3 min each) and infiltrated with a 1:1 mixture of ethanol and Durcupan ACM resin (Fluka) for 60 min with agitation, followed by three exchanges of 100% Durcupan resin (2 h each) with agitation. Resin was polymerized at 60°C for 48 h. Glass coverslips were removed, and ∼2 mm blocks were excised and mounted on dummy blocks. Ultrathin sections (∼70 nm) were cut using a Leica ultramicrotome and collected on 200‐mesh uncoated thin‐bar copper grids. Images were acquired using a Tecnai Spirit electron microscope (FEI) operated at 80 kV with a Gatan 2K × 2K CCD camera at 2.9 nm/pixel. For quantitative analysis, mitochondrial profile lengths and cross‐sectional areas were measured in ImageJ (National Institutes of Health). Mitochondrial number was normalized to the total RGC soma area per image. Mitochondrial volume density, defined as the ratio of mitochondrial volume to total cytoplasmic volume, was estimated stereologically.

### 4.15 SBEM Analysis

Retinal tissue for SBEM was prepared as previously described [39, 45, 70]. SBEM was performed on a Gemini scanning electron microscope (Zeiss) equipped with a 3View2XP stage and an OnPoint backscatter detector (Gatan). Volumes were manually segmented and reconstructed using IMOD software (http://bio3d.colorado.edu/imod/). Mitochondrial morphology metrics were quantified using Morpheme, a suite of Python and Amira scripts (https://github.com/CRBS/morphome). Distance measurements within SBEM‐derived volumes were performed using MTK commands in 3Dmod. Individual synaptic vesicles within the ribbon‐attached pool were manually labeled, and the shortest distance from each vesicle to the synaptic ribbon was calculated at nanometer resolution. The number of mitochondria located within defined distance ranges from the target synaptic ribbon (100–2000 nm) was similarly quantified.

### 4.16 Machine‐Learning‐Based Segmentation and Analysis

Machine‐learning‐based image segmentation was performed using CDeep3M, a cloud‐based deep learning segmentation tool [40, 41], and applied to serially sectioned SBEM datasets. A pretrained model was further refined through iterative training on large‐scale retinal SBEM data to enhance predictive confidence and model robustness. Multiple output layers, including enhanced SBEM images and predicted structural features, were integrated to generate 3D representations of cellular membranes, mitochondria, and synaptic vesicles. Segmented organelles from multilayer CDeep3M predictions were assembled into 3D structures using VAST‐lite [39]. Morphometric parameters, including synaptic bouton volume, vesicle number and volume, and mitochondrial surface area, volume, and length, were quantified using VAST‐lite in combination with MATLAB (MathWorks). Cellular structures and internal organelles were reconstructed from SBEM datasets using the IMOD software suite and Blender (version 2.81; https://www.blender.org). Synaptic ribbon bodies within presynaptic active zones were manually traced across serial SBEM sections and assembled into 3D models. Vesicles belonging to the ribbon‐attached pool and the proximal free pool [40] were annotated across serial sections and integrated into the corresponding synaptic ribbon reconstructions.

### 4.17 3D EM Tomography Analysis

3D EM tomography was performed on semi‐thick sections (∼350 nm) cut from cell blocks prepared for TEM as previously described [2, 39, 45]. 20‐nm colloidal gold particles were deposited on both sides of each grid to serve as fiducial markers for tilt‐series alignment. Prior to data collection, specimens were pre‐irradiated for ∼20 min at the acquisition magnification to minimize anisotropic specimen thinning during imaging. Dual‐axis tilt series were acquired using SerialEM software (University of Colorado) on a Tecnai Titan HiBase electron microscope (FEI) operated at 300 kV and equipped with a Gatan 4K × 4K CCD camera. Images were recorded at 0.81 nm/pixel. For each axis, 121 images were collected at 1° intervals over a tilt range of −60° to +60°; the grid was then rotated 90° and an orthogonal tilt series was acquired using identical parameters. To improve the signal‐to‐noise ratio, 2× binning was applied to each image by averaging 2 × 2 pixel boxes into single pixels using the newstack command in IMOD (University of Colorado; http://bio3d.colorado.edu/imod/). Tilt‐series alignment, tomographic reconstruction, and volume segmentation were performed using IMOD. Mitochondrial membranes were segmented by manual tracing in each 1.62 nm‐thick x‐y plane. Segmented contours were rendered as meshes for 3D model generation. Mitochondrial membrane surface areas and volumes were measured from segmented volumes using IMODinfo. Crista density, the ratio of total cristae membrane surface area to mitochondrial volume, was measured using ImageJ. Crista junctions were identified by placing spheres of equivalent diameter at each junction, and the average number of crista junctions per crista was expressed as the ratio of total crista junctions to total cristae per segmented mitochondrion. The opening size of each crista junction was measured along its long axis using 3Dmod.

### 4.18 Modeling the Rate of ATP Production From EM Tomography Volumes

The rate of mitochondrial ATP production was estimated using a biophysical modeling framework that incorporates electrochemical potential, proton concentrations, and proton‐motive force, and that also incorporates spatially accurate geometric representations of the cristae membrane, inner boundary membrane, and crista junctions, as previously described^39^. The parameter that most influences the model of mitochondrial ATP production is the portion of the curved cristae membrane where the ATP synthase molecules reside. The density of “highly” curved cristae is defined as the surface area of the curved portion of cristae divided by the mitochondrial volume.

### 4.19 OCR Analysis

OCR was measured as an index of mitochondrial respiration using a Seahorse XF96 Pro Analyzer (Agilent Technologies), as previously described [2, 69]. Primary RGCs were seeded into Seahorse XF96‐well plates at 1.5 × 10^4^ cells per well and transduced with either AAV2‐Null or AAV2‐AKAP1 for 3 days. Where indicated, cells were subsequently treated with 25 µm paraquat (PQ) for 6 h to induce oxidative stress prior to OCR measurement. Basal respiration was recorded first, followed by sequential injection of the following pharmacological inhibitors and uncouplers: oligomycin (2 µg/mL; Sigma–Aldrich), an ATP synthase inhibitor, to assess ATP‐linked respiration; carbonyl cyanide 4‐(trifluoromethoxy)phenylhydrazone (FCCP; 1 µm; Sigma–Aldrich), a mitochondrial uncoupler, to assess maximal respiration and spare respiratory capacity; and rotenone (2 µm; Sigma–Aldrich), a mitochondrial complex I inhibitor, to abolish mitochondrial respiration. OCR parameters, including basal respiration, maximal respiration, ATP‐linked respiration, and spare respiratory capacity, were derived from the resulting respiratory profile.

### 4.20 Quantitative PCR Analysis

Total RNA was extracted from isolated retinal tissue by homogenization in 1 mL TRIzol reagent (Invitrogen) followed by standard RNA isolation and purification, as previously described [39]. RNA was quantified, and 1 µg of total RNA was reverse‐transcribed into complementary DNA (cDNA) using the High‐Capacity RNA‐to‐cDNA Kit (Thermo Fisher Scientific) according to the manufacturer's instructions. Thermal cycling conditions were as follows: initial denaturation at 95°C for 5 min, followed by 40 cycles of 95°C for 30 s, 60°C for 30 s, and 72°C for 20 s, with a final extension at 75°C for 7 min. Relative mRNA expression levels were normalized to GAPDH and calculated using the 2^(−ΔΔCt) method. Quantitative real‐time PCR (qRT‐PCR) was performed using SYBR Green PCR Master Mix (Applied Biosystems) on a compatible real‐time PCR system. Mouse‐specific primer pairs for each target gene were designed using Primer‐BLAST (NCBI) and are listed in Table S5.

### 4.21 Cell Viability and Cytotoxicity Assays

Cell viability and cytotoxicity were assessed in parallel using MTT and LDH release assays, respectively, as previously described [39, 69]. Primary RGCs were transduced with either AAV2‐AKAP1 or AAV2‐Null for 3 days, then exposed to 50 µm PQ or elevated HP (30 mmHg) for 24 h, as described above. For the MTT assay, mitochondrial metabolic activity was assessed using the Cell Proliferation Kit I (MTT; Roche Diagnostics) according to the manufacturer's protocol. Following treatment, 10 µL of MTT stock solution was added to each well, including untreated controls, and cells were incubated for 3–4 h at 37°C in a humidified 5% CO_2_ incubator. Formazan crystals were dissolved by adding 100 µL of dimethyl sulfoxide (DMSO), and absorbance was measured at 560 nm with background correction at 690 nm using a SpectraMax microplate reader (Molecular Devices). For the LDH release assay, cell cytotoxicity was evaluated using a colorimetric LDH assay kit (MilliporeSigma) according to the manufacturer's instructions. Following the same treatment conditions, 50 µL of cell culture supernatant was transferred to a fresh 96‐well plate, and an equal volume (50 µL) of LDH reaction mixture was added. Samples were incubated in the dark at room temperature for 30 min, and absorbance was recorded at 490 nm with background correction at 680 nm using the same microplate reader. LDH release was expressed as a percentage of total cellular LDH content, reflecting the extent of plasma membrane damage. All assays were performed in triplicate (*n* = 3 replicate wells per experimental group).

### 4.22 ROS Measurement

Mitochondrial superoxide production was assessed using MitoSOX Red (Invitrogen) according to the manufacturer's protocol [69]. Primary RGCs were transduced with either AAV2‐AKAP1 or AAV2‐Null for 3 days, then exposed to 50 µm PQ for 24 h to induce oxidative stress, as described above. Following treatment, cells were co‐stained with MitoSOX Red (500 nm) and MitoTracker Green (100 nm; Invitrogen) for 30 min at 37°C in culture medium. Cells were then washed twice with PBS to remove excess dye. Fluorescence images were acquired using a Keyence BZ‐X810 All‐in‐One Fluorescence Microscope (Keyence Corp. of America) with identical acquisition parameters across all groups. MitoSOX Red fluorescence intensity was quantified using ImageJ (National Institutes of Health) and normalized to MitoTracker Green signal to account for differences in mitochondrial mass.

### 4.23 Statistical Analysis

All data are presented as mean ± SEM. Comparisons between two groups were performed using a two‐tailed unpaired Student's *t*‐test. For comparisons among three or more groups, one‐way analysis of variance (ANOVA) followed by Tukey's post hoc multiple comparisons test was used. For ultrastructural analyses (transmission EM, EM tomography, and SBEM), multiple mitochondria, presynaptic terminals, synaptic ribbons, or vesicles were measured per retina; these measurements were first averaged within each retina, and the retina (one per animal) was used as the experimental unit for statistical comparison, so that n denotes the number of animals rather than the number of structures. All statistical analyses were performed using GraphPad Prism. A P value of less than 0.05 was considered statistically significant. Statistical significance is denoted as ^*^ *p* < 0.05, ^**^ *p* < 0.01, ^***^ *p* < 0.001, and ^****^ *p* < 0.0001. Sample sizes (n) for each experimental group are reported in the corresponding figure legends.

## Author Contributions

**T.B. and W.‐K.J**. conceptualized the study, drafted the initial manuscript, and created figures. **T.B., K.‐Y.K., Z.S., G.A.P., M.P., Y.L., H.C., S.‐H.C., and W.‐K.J**. performed experiments and analyzed data. **T.B., K.‐Y.K., Z.S., G.A.P., M.P., Y.L., H.C., S.‐H.C., W.‐K.J**. analyzed data. **M.H.E. and R.N.W**. contributed to the discussion of data analysis and interpretation. **T.B., K.‐Y.K., G.A.P., M.H.E., R.N.W., and W.‐K.J**. wrote and revised the paper. All authors edited and approved the manuscript.

## Funding

This work was supported by the National Institutes of Health grants EY031697 (WKJ, GAP), EY034116 (WKJ, SHC, KYK), EY022589 (Vision core grant), Research to Prevent Blindness (New York, NY), University of California Gene Therapy Initiative (WKJ), and 5U01NS142013 (MHE).

## Ethics

Ethics approval for the study was obtained from the Institutional Animal Care and Use Committee at the University of California, San Diego (approval numbers: S12063 and S12067).

## Conflicts of Interest

The authors declare no conflicts of interest.

## Supporting information




**Supporting File 1**: advs78349‐sup‐0001‐SuppMat.docx.

**Supporting File 2**: advs78349‐sup‐0002‐MovieS1.mp4.

**Supporting File 3**: advs78349‐sup‐0003‐MovieS2.mp4.

**Supporting File 4**: advs78349‐sup‐0004‐MovieS3.mp4.

**Supporting File 5**: advs78349‐sup‐0005‐MovieS4.mp4.

**Supporting File 6**: advs78349‐sup‐0006‐MovieS5.mp4.

**Supporting File 7**: advs78349‐sup‐0007‐MovieS6.mp4.

**Supporting File 8**: advs78349‐sup‐0008‐MovieS7.mp4.

**Supporting File 9**: advs78349‐sup‐0009‐MovieS8.mp4.

**Supporting File 10**: advs78349‐sup‐0010‐MovieS9.mp4.

**Supporting File 11**: advs78349‐sup‐0011‐MovieS10.mp4.

**Supporting File 12**: advs78349‐sup‐0012‐MovieS11.mp4.

## Data Availability

The data that support the findings of this study are available on request from the corresponding author. The data are not publicly available due to privacy or ethical restrictions.
