# Supplementary material for: AKAP1 Protects Retinal Ganglion Cells and Preserves Vision by Improving Mitochondrial and Synaptic Health in Glaucomatous Neurodegeneration
Source: Adv Sci (Weinh). 2026 Oct 18:e78349. Online ahead of print. doi: 10.1002/advs.78349 (PMC13676990; doi:10.1002/advs.78349)
Supplement: Supplementary file 1 [file ADVS-9999-e78349-s001.docx]

**Supplementary Information for**

**AKAP1 Protects Retinal Ganglion Cells and Preserves Vision by Improving Mitochondrial and Synaptic Health in Glaucomatous Neurodegeneration**

Authors: Tonking Bastola^1^, Keun-Young Kim^2,^*, Ziyao Shen^1,^*, Guy A. Perkins^2^, Muna Poudel^1^, Yoonjin Lim^1^, Hyejeong Choi^1^, Soo-Ho Choi^3^, Mark H. Ellisman^2^, Robert N. Weinreb^1^, and Won-Kyu Ju^1,4,5^

**This Supplementary Information includes:**

Figures S1–8

Tables S1–5

Movie S1–11 Legends

**Figure S1. Decreased AKAP1 expression in non-treated D2 retinas with aging and AKAP1-His immunoreactivity in glaucomatous D2-AKAP1 retinas.** (*A*) IOP measurement in 3-, 5-, and 10-month-old non-treated D2 mice (*n* = 6 mice per group). (*B*) Representative image of a Western blot and densitometry graph for AKAP1 and RBPMS in non-treated D2 retinas with aging (*n* = 4 retinas from 4 mice per group). (*C*) Schematic representation of the recombinant AAV2-AKAP1 vector. The expression cassette consists of the CMV promoter driving murine Akap1 (1–857 amino acids; 1–2571 nt), followed by a C-terminal 6x-His tag and the human growth hormone (hGH) polyadenylation signal (1–478 nt). *(D)* Representative retinal images for AKAP1-His (green, arrows) and TUJ1 (red) immunoreactivities in the retinas. *(E)* Quantitative fluorescent intensity of His immunoreactivity in the GCL (*n* = 10 retina sections from 3 mice per group). Error bars represent SEM. Statistical analysis was performed using one-way ANOVA and Tukey’s multiple comparisons test. **P* < 0.05, ***P* < 0.01, ****P* < 0.001, and *****P* < 0.0001. Scale bar, 20 μm.

**Figure S2.** Representative image of a Western blot and densitometry graph for PKA expression in the retinas (*n* = 3 retinas from 3 mice per group). Representative retinal images for PKA (green) and RBPMS (red) immunoreactivities. Quantitative fluorescent intensity of PKA immunoreactivity in the GCL (*n* = 12 retina sections from 3 mice per group). Error bars represent SEM. Statistical analysis was performed using an unpaired Student's *t*-test. ***P* < 0.01. Scale bar, 20 μm.

**Figure S3. AKAP1 overexpression promotes RGC survival under elevated HP conditions.** (*A*) Experimental schematic and timeline of AAV2-AKAP1 or AAV2-AKAP1-GFP transduction in cultured primary RGCs and data analysis. (*B*) Representative images for AKAP1-GFP (yellow) expression in TUJ1(red)-labeled RGCs. (*C*) Western blot analysis for His, AKAP1, and RBPMS expression in cultured RGCs (*n* = 3 to 4 biological replicates per group). (*D*) Quantitative analyses of RGC viability and death using MTT and LDH release assays under elevated HP conditions (*n* = 12 biological replicates per group). Error bars represent SEM. Statistical analysis was performed using one-way ANOVA and Tukey’s multiple comparisons test. **P* < 0.05, ****P* < 0.001, and *****P* < 0.0001. Scale bars, 20 μm. Panel (A) includes graphical elements created by BioRender (www.biorender.com).

**Figure S4. AKAP1 overexpression protects RGCs against PQ-induced oxidative stress.** (*A*) Experimental schematic and timeline of AAV2-AKAP1 transduction and PQ (50 μM) treatment in cultured primary RGCs and data analysis. (*B*) Quantitative PCR analysis for *Akap1* gene expression in cultured RGCs under oxidative stress conditions (*n* = 3 biological replicates per group). (*C*) Western blot analysis for AKAP1 and RBPMS expression in cultured RGCs under oxidative stress conditions (*n* = 4 biological replicates per group). (*D*) Quantitative analyses of RGC viability and death using MTT and LDH release assays under oxidative conditions (*n* = 4 or 5 biological replicates per group). (*E*) OCR changes in AAV2-AKAP1-treated RGCs under PQ-induced oxidative stress conditions. (*F*) Quantitative analysis of OCR parameters: basal respiration, maximal respiration, ATP production, and spare capacity in cultured RGCs under oxidative stress conditions (*n* = 5 biological replicates per group). (*G*) Representative images for Mito-tracker green (green), MitoSOX red (red), and Hoechst (blue) staining in cultured RGCs under oxidative stress conditions. (*H*) Quantitative fluorescent intensity of MitoSOX (red) (*n* = 4 biological replicates per group). Error bars represent SEM. Statistical analysis was performed using one-way ANOVA and Tukey’s multiple comparisons test. **P* < 0.05, ***P* < 0.01, ****P* < 0.001, and *****P* < 0.0001. Scale bar, 20 μm. Panel (A) includes graphical elements created by BioRender (www.biorender.com).

**Figure S5. AKAP1 overexpression preserves mitochondrial dynamics and promotes ATP production in RGCs, thereby protecting against PQ-induced oxidative stress.** (*A*) Experimental schematic and timeline of AAV2-AKAP1 transduction and PQ (50 μM) treatment in cultured primary RGCs and data analysis. (*B*) Representative TEM images of mitochondrial morphology with 3D-reconstruction and cristae junction (arrowhead) of Null-CNT (a-f), Null-PQ (g-l), and AKAP1-PQ (m-r). (*C*) Measurements for mitochondrial profile length (*n* = 20 mitochondria per condition), mitochondrial profile area (*n* = 20 mitochondria per condition), number per unit area (*n* = 10 mitochondria per condition), volume density (*n* = 10 mitochondria per condition), cristae density (n=10 mitochondria per condition), rate of ATP generation per mitochondrial volume (*n* = 10 mitochondria per condition), number of crista junction per crista (n = 10 mitochondria per condition) and crista junction width (*n* = 20 mitochondria per condition). The number of replicates was 10 independent, sparsely spaced, primary RGCs, 5 each from 2 plates per condition for all measurements. Error bars represent SEM. Statistical analysis was performed using one-way ANOVA and Tukey’s multiple comparisons test. **P* < 0.05, ***P* < 0.01, ****P* < 0.001, and *****P* < 0.0001. Scale bars, 500 nm. Panel (A) includes graphical elements created by BioRender (www.biorender.com).

**Figure S6. Synapsin expression in glaucomatous D2 retinas.** (*A*) Western blot analysis for total synapsin expression in D2G-Null, D2-Null, and D2-AKAP1 retinas (*n* = 3 retinas from 3 mice per group). (*B*) Representative retinal images for total synapsin immunoreactivity in D2G-Null, D2-Null, and D2-AKAP1 retinas. (*C*) Quantitative fluorescent intensity of total synapsin immunoreactivity in the IPL of D2G-Null, D2-Null, and D2-AKAP1 retinas (*n* = 9 retinal sections from 3 mice per group). Error bars represent SEM. Statistical analysis was performed using an unpaired Student’s t-test or one-way ANOVA and Tukey’s multiple comparisons test. **P* < 0.05 and ***P* < 0.01. Scale bar, 20 µm.

**Figure S7. AKAP1 preserves PSD95 in glaucomatous D2 retinas.** (A) Western blot analysis for PSD95 expression in D2G-Null, D2-Null, and D2-AKAP1 retinas (*n* = 4 retinas from 4 mice per group). (*B*) Representative retinal images for PSD95 immunoreactivity in D2G-Null, D2-Null, and D2-AKAP1 retinas. (*C*) Quantitative fluorescent intensity of PSD95 immunoreactivity in the OPL, IPL, and inner retinal layer (IRL: IRL includes INL, IPL, and GCL) of D2G-Null, D2-Null, and D2-AKAP1 retinas (*n* = 9 retinal sections from 3 mice per group). Error bars represent SEM. Statistical analysis was performed using one-way ANOVA and Tukey’s multiple comparisons test. **P* < 0.05, ****P* < 0.001, and *****P* < 0.0001. Scale bar, 20 µm.

**Supplementary Figure 8. Schematic summary for retinal AKAP1 expression-mediated structural and functional preservation of mitochondria and synapses in the IPL of the glaucomatous retina.** Our findings identify retinal AKAP1 as a candidate regulator of mitochondrial and synaptic homeostasis and highlight AAV2-AKAP1 gene therapy as a promising neuroprotective strategy for vision preservation in glaucomatous neurodegeneration. The Schematic summary includes graphical elements created by BioRender (www.biorender.com).

**Supplementary Tables**

**Table S1.** Effect of AAV2-AKAP1 on RBPMS-positive RGC survival in the middle and peripheral retina from 10-month-old glaucomatous DBA/2J mice, related to Figure 1K.

| Strain (DBA/2J) | Treatment | Age  (Months) | Mean IOP (mmHg)  at age of 10 months | RGC density per retina  (RGCs/mm^2^) | |
| --- | --- | --- | --- | --- | --- |
|  | | | | Middle | Peripheral |
| D2G-Null | AAV2-Null | 10 | 10.7 ± 0.4 | 3427 ± 66 | 2853 ± 68 |
| D2-Null | AAV2-Null | 10 | 26.9 ± 0.6 | 1915 ± 197 | 1474 ± 138 |
| D2-AKAP1 | AAV2-AKAP1 | 10 | 26.5 ± 0.5 | 2883 ± 55 | 2360 ± 75 |

All results were reported as means ± SEM. *n* = 9-18 retina wholemounts from 5-15 mice per group.

**Table S2.** Effect of AAV2-AKAP1 on RBPMS-positive RGC survival in the middle/peripheral retina from glaucomatous mice induced by microbead (MB)-induced ocular hypertension, related to Figure 2C.

| Strain  (C57BL/6J) | Treatment | Age  (Months) | Mean IOP (mmHg)  at 8 weeks of MB | RGC density per retina  (RGCs/mm^2^) | |
| --- | --- | --- | --- | --- | --- |
|  | | |  | Middle | Peripheral |
| Null-CNT | AAV2-Null | 5 | 13 ± 0.4 | 3496 ± 70 | 2709 ± 72 |
| Null-MB | AAV2-Null | 5 | 26.2 ± 1 | 2805 ± 66 | 2318 ± 62 |
| AKAP1-MB | AAV2-AKAP1 | 5 | 26.4 ± 1.3 | 3503 ± 111 | 2849 ± 113 |
| AKAP1-CNT | AAV2-AKAP1 | 5 | 12.2 ± 0.5 | 3644 ± 54 | 2860 ± 41 |

All results were reported as means ± SEM. *n* = 7-10 retina wholemounts from 7-10 mice per group.

**Table S3.** Effect of AKAP1 loss on RBPMS-positive RGC survival in middle and peripheral retina from glaucomatous mice induced by microbead (MB)-induced ocular hypertension, related to Figure 2I.

| Strain (C57BL/6J and *Akap1^–/–^*) | Age  (Months) | Mean IOP (mmHg)  at 8 weeks of MB | RGC density per retina  (RGCs/mm^2^) | |
| --- | --- | --- | --- | --- |
|  | |  | Middle | Peripheral |
| WT-CNT | 5 | 13 ± 0.3 | 2861 ± 108 | 2569 ± 68 |
| WT-MB | 5 | 22.9 ± 0.5 | 2673 ± 70 | 2191 ± 57 |
| *Akap1^–/–^*-MB | 5 | 27.8 ± 1.3 | 2693 ± 84 | 2113 ± 68 |
| *Akap1^–/–^*-CNT | 5 | 13.1 ± 0.4 | 3122 ± 49 | 2673 ± 48 |

All results were reported as means ± SEM. *n* = 8 retina wholemounts from 8 mice per group.

**Table S4. Key Resources and Reagents**

| **REAGENT OR RESOURCES** | **SOURCE** | **IDENTIFIER** |
| --- | --- | --- |
| Antibodies | Supplier | Catalogue number |
| AKAP1 | Cell Signaling Technology | CST #5203 |
| AMPK | Cell Signaling Technology | CST #5831 |
| Phospho-AMPK | Cell Signaling Technology | CST #2535 |
| β-ACTIN | Millipore | MAB1501 |
| Active BAX | Santa Cruz Biotechnology | sc-23959 |
| BCL-xL | Santa Cruz Biotechnology | sc-8392 |
| CaN | BD Bioscience | #610259 |
| Phospho-CaMKII | Abcam | Ab5683 |
| C1q | Quidel Corporation | A301 |
| DRP1 | BD Biosciences | #611113 |
| Phospho-DRP1 S616 | Cell Signaling Technology | CST #3455 |
| Phospho-DRP1 S637 | Cell Signaling Technology | CST #4867 |
| GFAP | Invitrogen | 13-0300 |
| Anti-6X His tag | Abcam | Ab245114 |
| 6x-His Tag | Invitrogen | MB1-21315 |
| IBA1 | Wako Chemicals | 016-20001 |
| MFN1 | Abcam | ab57602 |
| MFN2 | Abcam | ab56889 |
| OPA1 | BD Biosciences | #612607 |
| OXPHOS | Invitrogen | #458099 |
| p38 | Cell Signaling Technology | CST #8690 |
| Phospho-p38 | Cell Signaling Technology | CST #4511 |
| PGC-1α | Santa Cruz Biotechnology | sc-13067 |
| PKA | Santa Cruz Biotechnology | sc-28315 |
| PSD95 | Abcam | Ab18258 |
| IBA1 | Wako | 019-19741 |
| RBPMS | Novus Biologicals | NBP2-20112 |
| Phospho-Synapsin | Cell Signaling Technology | CST 2311S |
| Synapsin | EMD Millipore | AB1543 |
| Synaptophysin | GeneTex | GTX633821 |
| TFAM | GeneTex | GTX77852 |
| TUJ1 | BioLegend | 801202 |
| Goat anti-rabbit HRP | Cell Signaling Technology | 7074 |
| Goat anti-mouse HRP | Cell Signaling Technology | 7076 |
| Alexa Fluor-488 conjugated donkey anti-mouse IgG antibody | Invitrogen | A-21203 |
| Alexa Fluor-568 conjugated donkey anti-mouse IgG antibody | Invitrogen | A-10037 |
| Alexa Fluor-488 conjugated donkey anti-rabbit IgG antibody | Invitrogen | A-21206 |
| Alexa Fluor-568 conjugated donkey anti-rabbit IgG antibody | Invitrogen | A-10042 |
| Alexa Fluor 594-conjugated CTB | ThermoFisher Scientific | C34777 |
| **Bacterial and Virus Strains** |  |  |
| AAV2-M4-AKAP1-His |  | N/A |
|  | | |
| **Chemicals, Reagent and Commercial Kits** | **Supplier** | **Catalogue number** |
|  |  |  |
| Ketamine | Ketaset, Zoetis Inc. | NDC 54771-2013 |
| MTT | Roche | 11465007001 |
| Mito Tracker Red | Invitrogen | M46752 |
| Paraformaldehyde | Sigma-Aldrich | P6148 |
| Paraquat | Sigma-Aldrich | 36541-100mg |
| Proparacaine | Sandoz Inc. | NDC 61314-016-01 |
| Seahorse XF Cell Mito Stress Test Kit | Agilent | 103015-100 |
| SuperSignal Chemiluminescent | Thermo Scientific | 34580 |
| Topical phenylephrine | Lifestar Pharma. | NDC 70756-649-35 |
| Tropicamide | Bausch & Lomb Incorporated | NDC 24208-590-64 |
| Xylazine | AnaSed LA, Cronus Pharma | NDC 13985-704-10 |
| Tissue Adhesive-Vetbond | 3M | 1469SB |
|  | | |
| **Experimental Models: Strains** | **Vendor** |  |
| C57BL/6J mice | Jackson laboratory | Stock No: 000664  RRID:IMSR_JAX:000664 |
| DBA/2J mice | Jackson laboratory | Stock No: 000671  RRID:IMSR_JAX:000671 |
| DBA/2J-*Gpnmb^+^*/SjJ mice | Jackson laboratory | Stock No: 007048  RRID:IMSR_JAX:007048 |
| AKAP1*^–/–^* mice |  |  |
| Sprague-Dalwley Rat | Envigo (Harlan) | N/A |
|  | | |
| **Software and Algorithms** | **Suppliers** | **Site Link** |
| ImageJ | NIH, USA | <https://imagej.nih.gov/ij/> |
| Adobe Photoshop | USA | <https://photoshop.com> |
| Prism 9 | GraphPad Inc. | <https://www.graphpad.com/scientific-software/prism/> |
| ROSALIND | Rosalind bio | <https://www.rosalind.bio/> |
| LatchBio | LatchBio | <https://latch.bio/> |

**Table S5. Primer list used in the experiment**

| SN | Primer list | Sequence (F) | Sequence (R) |
| --- | --- | --- | --- |
| 1 | AKAP1 | AAGCTATGACCCCACCACTG | CGCAACAGCTATCCACTGAA |
| 2 | Caspase1 | AGGAATTCTGGAGCTTCAATCAG | TGG AAA TGT GCC ATC TTC TTT |
| 3 | DRP1 | AGGTGGCCTTAACACTATTGACA | AGACGCTTAATCTGACGTTTGAC |
| 4 | GAPDH | AGAACATCATCCCTGCATCC | GTCCTCAGTGTAGCCCAAGA |
| 5 | IL-1β | GGAGAACCAAGCAACGACAAAATA | TGGGGAACTCTGCAGACTCAAAC |
| 6 | MFN1 | GTGAAGTTCACAAGTGCAAA | GCTCGGGTGGAGAAACTGCT |
| 7 | MFN2 | CCCTCGACAGTGTTTCTCCC | CCAGGCCAGTAACCATGGAG |
| 8 | OPA1 | CATCTACCTTCCAGCTGCCC | TCTCCTCCTTCACAGCCTCC |
| 9 | PGC-1α | AAGGTCCCCAGGCAGTAGAT | GCGGTATTCATCCCTCTTGA |
| 10 | NLRP3 | GCTCCAACCATTCTCTGACC | AAGTAAGGCCGGAATTCACC |
| 11 | TFAM | CCAAAAAGACCTCGTTCAGC | ATGTCTCCGGATCGTTTCAC |
| 12 | TNF-α | CATCTTCTCAAAATTCGAGTGAC | TGGGAGTAGACAAGGTACAACCC |

**Supplementary Movie Legends**

**Movie S1.** Null-NP, Surface-rendered mitochondrial volume after membrane segmentation, related to Figure 3M.

**Movie S2.** Null-EHP, Surface-rendered mitochondrial volume after membrane segmentation, related to Figure 3M.

**Movie S3.** Null-AKAP1, Surface-rendered mitochondrial volume after membrane segmentation, related to Figure 3M.

**Movie S4.** D2-Null, Surface-rendered mitochondrial volume and synaptic bouton after membrane segmentation, related to Figure 5J.

**Movie S5.** D2-AKAP1, Surface-rendered mitochondrial volume and synaptic bouton after membrane segmentation, related to Figure 5J.

**Movie S6.** D2-Null, Surface-rendered ribbon synapses, synaptic vesicles, and spinules in the synaptic bouton after membrane segmentation, related to Figure 6A.

**Movie S7**. D2-AKAP1, Surface-rendered ribbon synapses, synaptic vesicles, and spinules in the synaptic bouton after membrane segmentation, related to Figure 6B.

**Movie S8.** D2-Null, Surface-rendered synaptic ribbon and vesicles in the synaptic bouton, related to Figure 6G.

**Movie S9**. D2-AKAP1, Surface-rendered synaptic ribbon and vesicles in the synaptic bouton, related to Figure 6G.

**Movie S10.** D2-Null, Surface-rendered mitochondria and synaptic vesicles using CDeep3M and Vast Lite, related to Figure 7E.

**Movie S11.** D2-AKAP1, Surface-rendered mitochondria and synaptic vesicles using CDeep3M and Vast Lite, related to Figure 7E.
